# Quantification of promoting efficiency and reducing toxicity of Traditional Chinese Medicine: A case study of the combination of *Tripterygium wilfordii* hook. f. and *Lysimachia christinae* hance in the treatment of lung cancer

**DOI:** 10.3389/fphar.2022.1018273

**Published:** 2022-10-20

**Authors:** Xiaoyi Zhang, Kexin Wang, Hui Dai, Jieqi Cai, Yujie Liu, Chuanhui Yin, Jie Wu, Xiaowei Li, Guiyong Wu, Aiping Lu, Qinwen Liu, Daogang Guan

**Affiliations:** ^1^ Department of Biochemistry and Molecular Biology, School of Basic Medical Sciences, Southern Medical University, Guangzhou, China; ^2^ Guangdong Provincial Key Laboratory of Single Cell Technology and Application, Southern Medical University, Guangzhou, China; ^3^ Guangdong Provincial Key Laboratory on Brain Function Repair and Regeneration, Department of Neurosurgery, National Key Clinical Specialty/Engineering Technology Research Center of Education Ministry of China, Neurosurgery Institute, Zhujiang Hospital, Southern Medical University, Guangzhou, China; ^4^ Institute of Integrated Bioinformedicine and Translational Science, Hong Kong Baptist University, Hong Kong, Hong Kong SAR, China; ^5^ Hospital Office, Ganzhou People’s Hospital, Ganzhou, China; ^6^ Hospital Office, Ganzhou Hospital-Nanfang Hospital, Southern Medical University, Guangdong, China; ^7^ Guangdong-Hong Kong-Macau Joint Lab on Chinese Medicine and Immune Disease Research, Guangzhou, China

**Keywords:** *Tripterygium wilfordii* Hook. f., *Lysimachia christinae* Hance, lung cancer, network pharmacology, efficacy toxicity network, gene transmission chains

## Abstract

Traditional Chinese medicine (TCM) usually acts in the form of compound prescriptions in the treatment of complex diseases. The herbs contained in each prescription have the dual nature of efficiency and toxicity due to their complex chemical component, and the principle of prescription is usually to increase efficiency and reduce toxicity. At present, the studies on prescriptions have mainly focused on the consideration of the material basis and possible mechanism of the action mode, but the quantitative research on the compatibility rule of increasing efficiency and reducing toxicity is still the tip of the iceberg. With the extensive application of computational pharmacology technology in the research of TCM prescriptions, it is possible to quantify the mechanism of synergism and toxicity reduction of the TCM formula. Currently, there are some classic drug pairs commonly used to treat complex diseases, such as *Tripterygium wilfordii* Hook. f. with *Lysimachia christinae* Hance for lung cancer, *Aconitum carmichaelii* Debeaux with *Glycyrrhiza uralensis* Fisch. in the treatment of coronary heart disease, but there is a lack of systematic quantitative analysis model and strategy to quantitatively study the compatibility rule and potential mechanism of synergism and toxicity reduction. To address this issue, we designed an integrated model which integrates matrix decomposition and shortest path propagation, taking into account both the crosstalk of the effective network and the propagation characteristics. With the integrated model strategy, we can quantitatively detect the possible mechanisms of synergism and attenuation of *Tripterygium wilfordii* Hook. f. *a*nd *Lysimachia christinae* Hance in the treatment of lung cancer. The results showed the compatibility of *Tripterygium wilfordii* Hook. f. and *Lysimachia christinae* Hance could increase the efficacy and decrease the toxicity of lung cancer treatment through MAPK pathway and PD-1 checkpoint pathway in lung cancer.

## 1 Introduction

### 1.1 Background

Lung cancer is a malignant tumor originating from the bronchial mucosa or glands in the lung, with the fastest growth in morbidity and mortality. In recent years, the incidence rate and mortality of lung cancer have increased significantly. Specifically, the incidence rate ranks first in male malignant tumors and second in female malignant tumors (Wu et al., 2021). Lung cancer involves a complicated cascade change process, which is mediated by smoking, genetics, air pollution, and other factors. The treatments for lung cancer mainly include surgery, radiation therapy, immunotherapy, and chemical therapy. The selection of a specific treatment strategy needs to be determined by combining the systemic, and cardiopulmonary conditions of the patient and the stage of cancer development. However, there are still some unsolved issues in the existing schemes for the clinical treatment of lung cancer. Surgical treatment has the best effect, but only 20%–30% of patients are suitable for surgical treatment. Chemotherapy is the most widely used and mature treatment at present. However, it has severe clinical side effects and poor prognosis. As a local treatment, radiotherapy has little effect on metastatic tumors with obvious side effects. Therefore, in recent years, TCM has been widely used in the treatment of lung cancer because of its minor side effects and immunomodulatory advantages. Previous reports have confirmed that TCM could effectively inhibit angiogenesis, invasion and migration in lung cancer to reduce the adverse reactions, improve the curative effect, and the life quality of patients. Previous studies have shown that Yiqi Tongluo Jiedu Decoction combined with gefitinib may inhibit the growth of BALB/c nude mice xenografts by up-regulating and down-regulating the expression of Caspase-3 and VEGF, respectively, and also can inhibit the tumor angiogenesis (Yan, 2017). Jiajian Shashen Maidong decoction integrated with paclitaxel and cisplatin can significantly improve the immune function of patients with lung cancer undergoing chemotherapy and improve the quality of life of patients with lung cancer (Xiao-su, 2011). Apart from assisting western medicine in treattumorsumor, TCM formula can also independently play an anti-cancer role. Berberine could diminish the expression of PD-L1 in lung cancer cells and facilitates antitumor immunity *via* inhibiting the deubiquitination activity of CSN5 (Liu et al., 2020). Maimendong and Qianjinweijing Tang (Jin formula) inhibit the proliferation, migration and invasion of A549 and H1299 cells by up-regulating miR-149–3p and down-regulating the Wnt/β-catenin signaling pathway, achieving the effect on cancer inhibition (Jiang et al., 2020). In addition, in clinical trials, Yiqi Yangyin Jiedu Decoction has been proved to be ameliorate the qi-yin deficiency syndrome evidently in advance lung cancer patients, and the Karnofsky score is significantly higher than that of chemotherapy group, improving the quality of life of lung cancer patients. At the same time, it shows a good clinical effect when combined with chemotherapy (Liu Lingshuang et al., 2008). Compared with chemotherapy group, Fei Fufang for treating senile non-small cell lung cancer, especially for the patients with mid-stage or late-stage lung cancer, can relieve clinical symptoms, improve quality of life, increase weight, stabilize tumor, delay disease progression and prolong survival time (Wei, 2012). It is suggested that TCM is one of the safe and effective treatments for lung cancer. Some TCM with anticancer properties also has the function of treating lung cancer. For example celastrol could suppress the proliferation and induce the apoptosis of A549 cells through the mitochondrial pathways (Zhiyang3, 2018). Therefore, TCM combined with western medicine has a wide application prospect in the treatment of lung cancer.

Herb pairs is the simplest and most widely used prescription form in the process of treating complex diseases in TCM. The main compatibility purpose of TCM pairs is to increase effectiveness and reduce toxicity. At present, some TCM pairs are widely used in the treatment of diseases such as *Prunus armeniaca* L. with *Glycyrrhiza uralensis* Fisch.; *Astragalus aaronii* (Eig) Zohary with *Atractylodes amurensis* (Freyn ex Kom.) H.S.Pak; *Notopterygium forrestii* H. Wolff with *Heracleum hemsleyanum* Diels; *Lysimachia christinae* Hance with *Forsythia suspensa* (Thunb.) Vahl; and *Coptis chinensis* Franch. With *Isatis afghanica* Hadač and Chrtek. Among these anti-tumor drug pairs, there are also some frequently used herb pairs such as *Tripterygium wilfordii* Hook. f. with *Glycyrrhiza uralensis* Fisch., *Pteris multifida* Poir., and *Lysimachia christinae* Hance; *Paeonia lactiflora* Pall. With *Tripterygium wilfordii* Hook. f.; *Aconitum carmichaelii* Debeaux is mixed with *Glycyrrhiza uralensis* Fisch., *Rheum officinale* Baill., and *Panax notoginseng* (Burkill) F.H.Chen*.*

Among them, *Tripterygium wilfordii* Hook. f. (LGT) and *Lysimachia christinae* Hance (JQC) are one of the commonly used compatibility pairs for tumor treatment. The diterpenoids and triterpenoids of LGT and the flavonoids and polysaccharides in JQC have anti-tumor effects. Previous pharmacological studies found that the alcohol extracts of LGT and JQC had a synergistic effect on the inhibition of cell proliferation of non-small cell lung cancer. JQC combined with LGT could exert a synergistic effect and reduce the toxicity of LGT on lung cancer with the best effect at the concentration ratio of 2/1 (Jun-Ming et al., 2016). In S180 mice, the combination of LGT and JQC could significantly reduce the hepatotoxicity and nephrotoxicity caused by LGT promote the expression of Nrf2 pathway and improve the tumor inhibition rate (Wang et al., 2018). In addition, some pharmacological experiments showed that the increased serum alanine/aspartate aminotransferase (ALT/AST) level of LGT could be significantly reversed by the compatibility with JQC to achieve the detoxification effect (Wang et al., 2018). LGT compatibility with JQC could significantly decrease the levels of pro-inflammatory cytokine tumor necrosis factor-alpha and malondialdehyde, while anti-inflammatory cytokine interleukin (IL)-10 and glutathione levels all increased in livers and kidneys of mice. It indicates that the toxicity-reduced mechanism of LGT compatibility with involves inhibiting hepatic and kidney oxidative stress and inflammation (Wang et al., 2019a). In addition, the compatibility of LGT and JQC can reverse the high toxicity of LGT by affecting the liver and kidney metabolism of LGT. The levels of glutathione and glutathione s-transferase, glutathione peroxidase, superoxide dismutase and catalase in the liver and kidney of S180 tumor-bearing mice were significantly decreased, and LGT alone significantly reduced the above indexes, while JQC combined with LGT could significantly reverse the excessively low levels of the above indexes, which indicated that LGT combined with JQC could reverse the anti-oxidation level of LGT-reduced liver and kidney. At the same time, the combination of LGT and JQC can also significantly reduce the levels of alanine/aspartate transaminase, creatinine and urea nitrate in the serum of S180 tumor-bearing mice (Wang et al., 2019b). In addition, the combination of LGT and JQC can significantly reduce AUC and C_max_ of triptolide, and significantly increase the clearance rate, which indicates that the attenuation mechanism of the compatibility may reduce the tissue damage by accelerating the metabolism and excretion of triptolide, and reducing the tissue distribution concentration (al, 2015).

Although LGT and JQC play a therapeutic role in diseases by increasing efficiency and reducing toxicity, the specific mechanism of promoting efficiency and reducing toxicity of LGT and JQC is still no specific clarification in a quantitative way due to active components of LGT and JQC are act as multiple targets and multiple pathways on complex diseases. Therefore, it is necessary to find a suitable method to quantify these modes of increasing efficiency and reducing toxicity. Network analysis of multimodal data is one of the commonly used research strategies to analyze the treatment of complex diseases with TCM, such as the multi-component and multi-target mechanism of TCM prescriptions, the mechanism of “different treatments for the same disease” and “different diseases with the same treatment,” etc. However, there are few related research reports on the synergistic and attenuated mechanism of TCM compatibility rule based on network analysis.

At present, some studies have also reported the potential mechanism of increasing efficacy and reducing toxicity of herb pairs, such as the compatibility of *Glycyrrhiza uralensis* Fisch. and *Aconitum carmichaelii* Debeaux can not only exert the effect of strengthening heart and promoting blood pressure, but also antagonize the arrhythmia caused by *Aconitum carmichaelii* Debeau*x* by inhibiting the Na^+^ channel of myocardial cells (YAN G Ming and HU, 2003); *Aconitum carmichaelii* Debeaux and *Zingiber acuminatum* Valeton mainly focuses on strengthening the heart and reducing cardiac toxicity (Weiping, 2008). Most of these reports are experiment-driven, or the effect network and toxicity network are analyzed separately. The crosstalk and propagation influence characteristics of the effective-toxicity network are not considered comprehensively. In the process of herb pair’s therapeutic effect, the related monomers will have certain toxic and side effects and even interfere with the transmission of the effect. Therefore, how to integrate and analyze the efficacy and toxicity network is one of the key ways to accurately understand the compatibility of TCM prescriptions.

In this research, in order to quantitatively determine the synergy and attenuation mechanism of LGT and JQC, we design a new model, which integrates matrix decomposition and shortest path propagation, taking into account both the crosstalk of the effective network and the propagation characteristics. With the integrated model strategy, we can quantitatively detect that the possible mechanism of promoting efficiency and reducing toxicity of herb pairs in the compatibility process of TCM prescriptions.

## 2 Materials and methods

### 2.1 Flowchart

**FIGURE 1:**
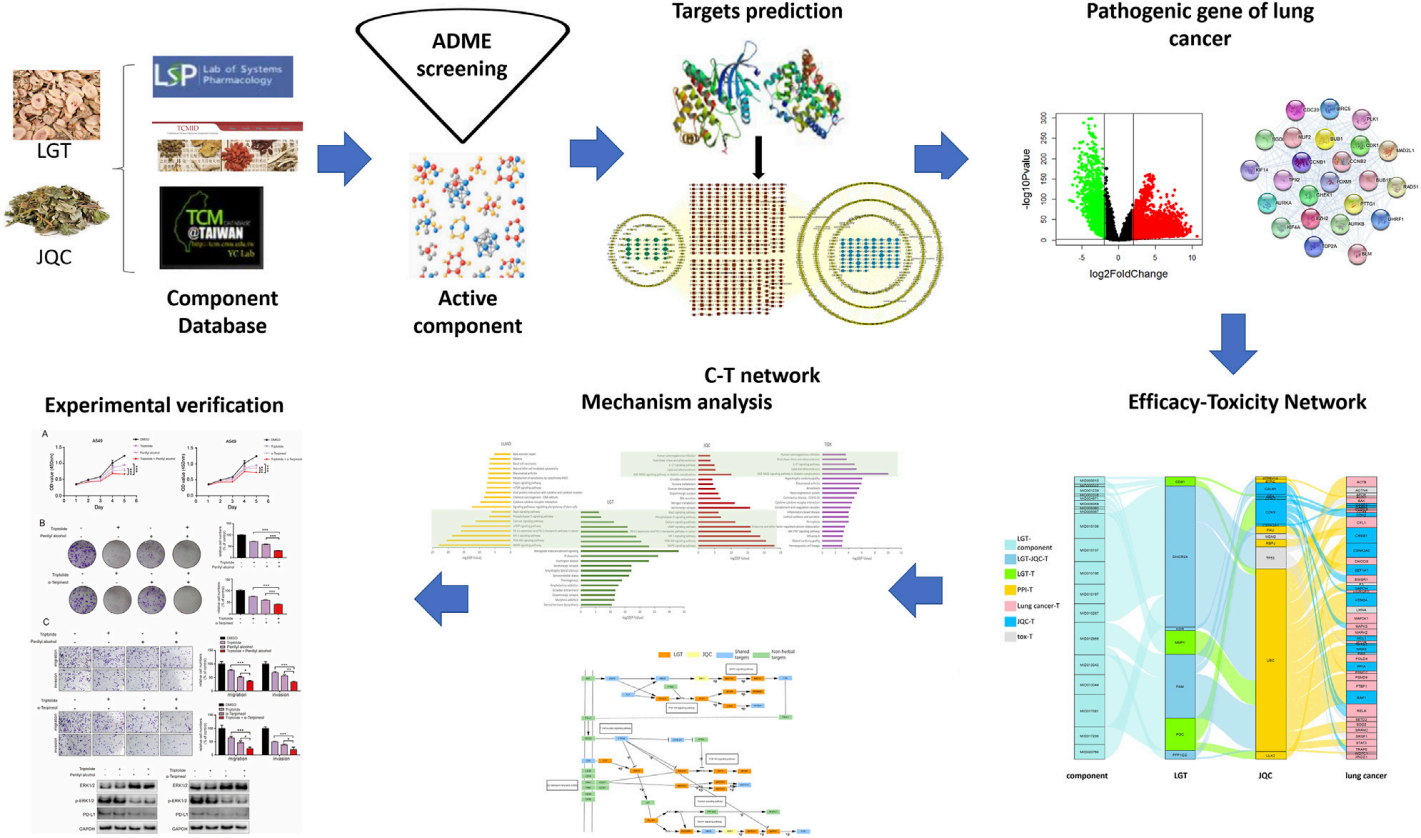
The flowchart scheme of our proposed quantitative integration method of dual efficacy and toxicity. LGT stands for *Tripterygium wilfordii* Hook. f., JQC stands for *Lysimachia christinae* Hance.

TCM compatibility therapy is widely used in clinical application, but the mechanism of promoting efficiency and reducing toxicity of herb pair has not been analyzed by system biology. In this study, we designed an integrated analysis model of efficacy-toxicity network model to explore the possible mechanism of the combination of LGT and JQC in the treatment of lung cancer, so as to provide reference for the clinical use of LGT and JQC in the treatment of lung cancer. The work flow is shown in Figure 1 and described as follows: 1) Collecting and sorting out the components of LGT and JQC from TCMSP, TCMID and TCM@Taiwan; 2) Sorting out the main active components of LGT and JQC based on published ADMET screening method; 3) Predicting the targets of active components in LGT and JQC; 4) Calculating the initial influence of active components on their targets. 5) Integrating the active ingredients in LGT and JQC, their targets and the pathogenic genes of lung cancer to construct the quantitative efficacy-toxicity network of LGT and JQC; 6) Using the common pathway among JQC, LGT and lung cancer to decipher the potential mechanism of promoting efficiency and reducing toxicity of LGT combined with JQC in treating lung cancer; 7) Experimental verification.

### 2.2 Collection and arrangement of LGT and JQC components

All chemical components of LGT and JQC were obtained from the TCM System Pharmacology Database and Analysis Platform database (TCMSP) (Ru et al., 2014) (http://lsp.nwsuaf.edu.cn/tcmsp.php), Traditional Chinese Medicine integrated database (Xue et al., 2013) (TCMID, http://www.megabionet.org/TCMID/) and TCM@Taiwan (Tsai et al., 2011) (http://tcm.cmu.edu.tw/zh-tw). The chemical identification and concentrations of LGT and JQC was collected from previous literature reports. All chemical structures were prepared and converted to standardized SMILES using the Open Babel Toolkit (Version 2.4.1).

### 2.3 Active component analysis of LGT and JQC

ADMET includes pharmacokinetic and toxicological issues such as whether a compound can be effectively absorbed by the human body and reach the target tissue, or whether a compound have toxicity, ADMET property usually list as key indicators for evaluating the ability of small molecular compounds to be made into pharmaceuticals (Lucas et al., 2019). Components passed the filtration of ADMET screening usually have better pharmacokinetic characteristics and higher bioavailability during *in vivo* metabolism, thus effectively reflecting the safety and effectiveness levels of candidate drugs and improving the success rate of drug research and development (Gu et al., 2021). In this study, the active components in LGT and JQC were primarily selected by ADEMT. The screening requirements of ADEMT include molecular weight less than 500 Da, number of hydrogen bond donors less than 5, number of hydrogen bond receptors less than 10, number of rotatable bonds less than 10, high gastrointestinal absorption and exclusion of components with high risk of inhibiting potassium channel (hERG_inhibition), etc.

### 2.4 Target prediction for LGT and JQC

Component target (C-T) networks for LGT and JQC were constructed using Cytoscape software (Version 3.9.1) (Lopes et al., 2010). The topological parameters of networks were analyzed using Cytoscape plugin NetworkAnalyzer (de Jong et al., 2003).

### 2.5 Constructing initial influence coefficient of components on their targets by matrix decomposition

The initial influence coefficient between the LGT components and the target was obtained by matrix decomposition. The matrix decomposition steps are as follows:

1. Perform a rank-L Eckart–Young–Mirsky approximation of A by singular value decomposition (SVD) as $A_{L}=U_{L}\sum_{L}V_{L}^{T}$ , and let $W=U_{L}$ .
2. Estimation of Y :

For i=1,…,M

Re-order rows of W so that the ith column of Y has the structure $y_{i}=\left[\begin{array}{c} {\tilde{y}}_{i}\\ 0 \end{array}\right]$ .

Partition $W=\left[\begin{array}{c} W_{c}\\ W_{r} \end{array}\right]$ conformally with this structure.

Do SVD for $W_{r}$ and get the last L-M+1 right singular vectors, denoted as a matrix $X_{0}$ . Compute ${\tilde{y}}_{i}=$ the first left singular vector of $W_{c}X_{0}X_{0}^{T}$ end.

3. Estimate the initial influence coefficient matrix by S = $Y^{†}A_{L}$

For the choice of L, it has been shown by published literature that we can choose L = M with good performance and superior computational efficiency (Chang et al., 2008). If L = M, due to the column dimension of $W_{r}$ (which is M) V0 in ${\hat{P}}_{{\hat{S}}_{r}^{T}}^{⊥}=X_{0}X_{0}^{T}$ becomes a 1-dimensional vector x, which is the Mth (the last) right singular vector of $W_{r}$ , so that

$${\tilde{P}}_{{\tilde{S}}_{r}^{T}}^{⊥}=xx^{T}$$

Then

$${\tilde{W}}_{c}={\tilde{P}}_{{\tilde{S}}_{r}^{T}}^{⊥}W_{c}^{T}=xx^{T}W_{c}^{T}={\bar{\tilde{s}}}_{1}{\tilde{y}}_{1}^{T}+{\bar{\Gamma }}_{0}^{T}$$

Γ is the inevitable measurement noise. Hence we get an estimate of ${\tilde{y}}_{1}$ simply as

$${\tilde{y}}_{1}=W_{c}$$

### 2.6 Identification of toxic targets

Through published literature retrieval, toxicity information of LGT was obtained, including which systems caused which specific toxic and side effects. For example, LGT has renal toxicity, which is likely to cause renal failure and renal insufficiency. The corresponding gene targets for toxic and side effects were obtained in the DisGeNET database (Pinero et al., 2021) (https://www.disgenet.org/) as toxicity targets for LGT.

### 2.7 Scoring of efficacy-toxicity network by using propagation model

The process of component transmission to the pathogenic gene through the target involves the effective targets and toxicity targets of the component. In this study, we constructed the efficacy-toxicity network using the propagation model. The principles of the propagation model are as follows:

(1) At the beginning, P={u},Q=H−{u} . For all nodes x in Q, if there is a path from u to x, then $d_{u,x}=c_{u,x}$ ; otherwise, $d_{u,x}=\infty$ .
(2) For all nodes x in Q, find the node t with smallest $d_{u,t}$ ,i.e.,:

$$d_{u,x}=\min\left\{d_{u,x}|x\in Q\right\}$$

$d_{u,t}$ is the shortest distance from target u to key lung cancer metabolic gene t. Node t is also the closest node to u among all nodes in Q. Delete node t from Q and merge it into P.

(3) Update the value of $d_{u,x}$ with the following formula for nodes x adjacent to t in Q

$$d_{u,x}=\min\left\{d_{u,x},d_{u,t}+c_{t,x}\right\}$$

(4) Continue the above steps until Q is an empty set

Therefore, we used the model to construct Gene Transmission Chains (GTC) against LGT component targets, Protein-protein interactions (PPI) networks, and pathogenic genes of lung cancer. And finally obtaining a GTC which is transmitted to a lung cancer pathogenic gene from a LGT component target through a plurality of genes:

A−−B−−C−−D−−E

In the GTC, we believe that the later the toxic target appears in the GTC, the less toxicity will be accumulated, and the earlier the JQC target appears, the better the synergistic effect will be. Therefore, we labeled the genes in GTC, with the component target of JQC in GTC labeled [JQC], and the toxicity target of LGT in GTC labeled [TOX]:

A−−B[JQC]−−C[TOX]−−D−−E

Using the lung cancer gene expression profile data obtained by The Cancer Genome Atlas (TCGA) (Tomczak et al., 2015) (https://portal.gdc.cancer.gov/), the correlation between the two genes was calculated and recorded as $cor\left(i,j\right)=s_{i,j}$ :

$$A-s_{A,B}-B\left[JQC\right]-s_{B,C}-C\left[TOX\right]-s_{C,D}-D-s_{D,E}-E$$

The initial value $v_{0}$ of the GTC is equal to the sum of the initial influence coefficients a corresponding to the first target of the GTC:

$$v_{0}=\sum a_{A_{k}}\left(k=1,\ldots ,n\right)$$

The initial score value $v_{1}$ of the GTC is equal to $v_{0}$ multiplied with the correlation between genes $s_{i,j}$ until the end of the GTC:

$$v_{1}=v_{0}s_{A,B}s_{B,C}s_{C,D}s_{D,E}$$

$$v_{1}=\sum v_{0}s_{i,j}$$

After the scores of complete GTCs were calculated, the change in scores when toxic targets and JQC targets were encountered in the GTCs was calculated. When toxic targets were encountered in the GTC, the scoring method was changed, and the scoring value was recorded as $v_{2}$ :

$$v_{2}=v_{0}s_{A,B}s_{B,C}s_{C,E}$$

The scoring method also changed when JQC targets were encountered in the GTCs, and the score value was recorded as $v_{3}$ :

$$v_{3}=v_{0}s_{A,B}s_{B,E}$$

Final score v of the GTC:

$$v=v_{1}-v_{2}+v_{3}$$

After the final score of complete GTCs was calculated, the median score $\left(\tilde{v}\right)$ of all GTCs was taken and all GTCs were screened. The GTCs with the score greater than $\tilde{v}$ were considered to be the GTCs with significant effect and were retained.

### 2.8 Reverse screening of key components for experimental verification

In order to screen out the components of LGT and JQC with the strongest synergistic effect, we calculated the synergistic scores of LGT and JQC according to the following steps:

(1) The number of targets of LGT component A is a , the number of targets of JQC component B is b , and the number of toxic targets of LGT component A is c . The score calculation formula is as follows:

$$s_{1}=\frac{a\cap b-c}{a+b}$$

(2) Scale the scores $s_{1}$ calculated by all paired components to the range of [0,1] to get $s_{2}$ .
(3) Through the common C-T network diagram of LGT-JQC, get the values of between for each component, which are denoted as x(A),y(B) , and the calculation formula is as follows:

$$s_{3}=\sqrt{x\times y}$$

(4) Final score s between paired components:

$$s=s_{2}+s_{3}$$

Details of all LGT and JQC pairing components and scores are recorded in Supplementary Table S1. By screening the high-scoring paired components, we selected Perillyl alcohol, Triptolide, and α-Terpineol as the components for experimental verification.

### 2.9 GO analysis and pathway analysis

To analyze the main functions of the GTCs, Gene Ontology (GO) analysis was performed using the Diversity Visualization Integrated Database (DAVID 6.8) (Huang da et al., 2009). And the pathway data were obtained from the Kyoto Encyclopedia of Genes and Genomes (KEGG) database for KEGG (Draghici et al., 2007) pathway enrichment analyses. *p*-values were set at 0.05 as the cut-off criterion. The results of analysis were annotated by Pathview (Luo and Brouwer, 2013) in the RBioconductor package (https://www.bioconductor.org/).

### 2.10 Experimental verification

#### 2.10.1 Cell culture and reagents

The human lung cancer cell A549 and the human hepatocyte cell L-02 were cultured in Dulbecco’s Modified Eagle’s Medium (Invitrogen, Shanghai, China) supplemented with 10% FBS (FSP500, ExCell bio, Shanghai), 100 U∙mL−1 penicillin and 100 μg mL−1 streptomycin at 37°C in 5% CO_2_ atmosphere. Triptolide, Perillyl alcohol and α-Terpineol (≥98% purity by HPLC) were purchased from Jingzhu Biotechnology (Nanjing, China) and dissolved in dimethyl sulfoxide (Sigma, United States).

#### 2.10.2 Cell viability assay

Cells viability was evaluated by Cell Counting Kit-8 (CCK-8; Selleck Chemicals, China). Briefly, A549 cells were digested and plated into each well of a 96-well plate after adjusting the cell suspension to the appropriate concentration of 1×10^3^ cells per 100 μL. The next day, the culture medium was discarded and the cells were exposed to Triptolide, Perillyl alcohol and α-Terpineol at different concentrations for 0 h, 24 h, 48 h, 72 h, 96 h and 120 h, 10 μL of CCK-8 (Bimake, United States) was added in the plates and cultured at 37 °C for 2 h. Absorbance at 450 nm was measured using spectrophotometer. The experiments were carried out in triplicate.

#### 2.10.3 Colony formation assay

1,000 cells were seeded in 6-weel plates with 2 ml complete DMEM. Then, new complete DMEM containing different concentrations of drugs was added. The medium should be replaced every 3–4 days. After 2 weeks of cultivation, the supernatant was abandoned and the cells were washed with phoshate buffered saline (PBS), then fixed with methanol and stained with crystal violet (Beyotime, China, C0121) for 15 min. The numbers of colonies were counted for analysis.

#### 2.10.4 Migration and invasion assays

Cells resuspended in FBS-free medium which containing different concentrations of drugs was placed into the upper uncoated chamber for migration assay or chamber coated with matrigel (BD Biosciences, Bedford, MA) for invasion assay. All the bottom chambers were added with 20% FBS medium. Following 48 h of incubation, cells remaining in the upper membrane were removed, whereas the migrated cells were fixed with methanol and stained with crystal violet, and then counted under the microscope.

#### 2.10.5 Western blot analysis

Cells were collected and total protein was extracted by RIPA lysis buffer (Beyotime, Shanghai, China, P0013 B), and BCA protein assay kit (Thermo Fisher Scientific, United States) was used to determine protein concentration. Equal amount of protein samples was resolved by SDS‐PAGE gel and the proteins were subsequently moved to PVDF membrane (Millipore, Bedford, MA), then the PVDF membrane were blocked (Beyotime, Shanghai, China, P0252) and immunoblotted with primary antibody of p44/42 MAPK (Erk1/2) (1:1,000; cat. no.4695T; Cell Signaling Technology), p-p44/42 MAPK (Erk1/2) (1:1,000; cat. no.4370T; Cell Signaling Technology), PD-L1 (1:1000, cat. no.66248-1-Ig, proteintech), GAPDH (1:1000, cat. no.60004-1-Ig, proteintech) at 4°C overnight. After washing by TBST (TBS containing 1% Tween), The membranes were exposed to corresponding secondary antibodies for 1 h. Antibody signal was detected using Clarity Western ECL substrate (Abbkine Scientific, China). The GAPDH served as an endogenous reference.

## 3 Results

### 3.1 Chemical analysis

Chemical component analysis plays an important role in the compatibility and the material basis research of Chinese material medica, and is the foundation of the research on the action mechanism of Chinese medicine. Through literature retrieval, we collected the high-concentration chemical components verified by HPLC and other experimental methods in LGT and JQC (Table 1). The chemical components of Chinese herbal medicine and the identified component concentrations provide auxiliary chemical experimental proof for searching the active components of the Chinese herbal medicine. This will provide a valuable reference for further analysis.

**TABLE 1:** Experimentally confirmed high concentration composition of LGT and JQC.

Herb	Method	Component	Concentration	References
*Tripterygium wilfordii* Hook.f	HPLC	Triptolide	677 μg/g	(Li and Wang, 2005; Cai et al., 2011; Wu et al., 2017; Guo et al., 2019)
		Wilfortrine	1601.3 μg/g	
		Triptophenolide	108.3 μg/g	
		Wilfordine	90.9 μg/g	
		Wilforgine	2987.5 μg/g	
		Triptonolide	15.3 μg/g	
		Wilforine	430.4 μg/g	
		Demethylzeylaseral	7769.3 μg/g	
		Celastrol	7732.5 μg/g	
		chlorogenic acid	250 μg/g	
*Lysimachia christinae* Hance	HPLC	quercetin	124 μg/g	(Guo et al., 2012; Jeong et al., 2021)
		Kaempferol	156 μg/g	
		Kaempferol-3-O-β-D-glucopyranoside	195 μg/g	
		rutin	146 μg/g	
		linarin	105 μg/g	
	GC-MS	perillyl alcohol	1.407% (Relative percentage of mass)	HOU Dong-yan, (2003)
		α-terpineol	0.767% (Relative percentage of mass)	
		Pulegone	0.376% (Relative percentage of mass)	
		alpha-humulene	0.947% (Relative percentage of mass)	

### 3.2 Component collection and active component screening in LGT and JQC

**FIGURE 2:**
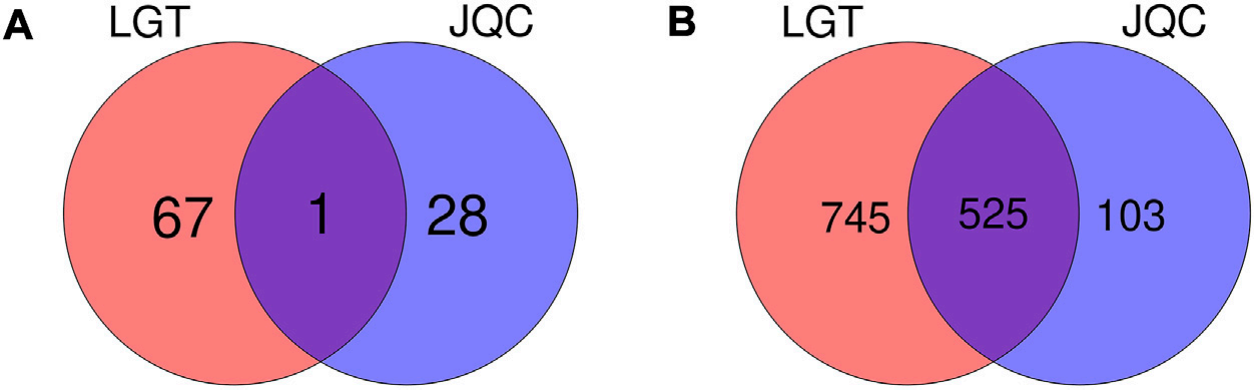
Venn diagram of components and targets of LGT and JQC. **(A)** Common and specific components of LGT and JQC. **(B)** Common and specific targets for LGT and JQC.

A total of 144 components in LGT and 61 components in JQC were obtained by combined searching of the TCMSP, TCMID, and TCM@Taiwan databases. ADMET screening is one of the commonly used filtrate methods for drug selection in pharmaceutical research. After ADMET screening, 68 active ingredients in LGT and 29 active ingredients in JQC passed the integrated filtering criteria (Table 2). Further analysis of the active components in LGT and JQC revealed that the LGT and JQC have 67and 28 unique components, respectively (Figure 2A). In addition, LGT and JQC have one component in common. The shared active components Kaempferol has many pharmacological effects, such as anti-cancer, anti-oxidation, anti-virus, anti-inflammation, anti-bacteria, and enhancing the immunity (Imran et al., 2019). Meanwhile, it can protect against LGT-induced acute liver injury (WANG Jun-ming et al., 2013). Triptolide, as one of the most important active ingredients of LGT, has immunosuppressive, anti-inflammatory, anti-fertility and anti-tumor biological activities. At the same time, it has different degrees of toxicity to heart, liver, bone marrow and spleen (Mingxing Liu et al., 2005). Triptolide also has anti-tumor effect *in vitro* and induces apoptosis. Among the components of JQC, perilla alcohol could sensitize lung tumor cells to apoptosis by genetic lesions present in tumor cells (Xu et al., 2004). In addition, as the ethanol extract component of JQC, quercetin could protect the liver injury induced by LGT by reducing lipid peroxidation in mouse liver and enhancing the activities of superoxide dismutase and catalase (WANG Jun-ming et al., 2013). These results suggest that the compatibility of LGT and JQC may exert the efficacy of enhancing the efficacy and reducing the toxicity in the treatment of lung cancer by affecting the common and specific components.

**TABLE 2:** Components in LGT and JQC for further analysis after screening by ADME.

ID	Component	MW	HDON	HACC	RBN	Fraction Csp3	GI absorption	hERG_inhibition	Source
MID002175	Dehydroabietlc acid	300.44	1	2	2	0.65	High	low_risk	*Tripterygium wilfordii* Hook.f
MID002604	1,8-Dihydroxy-4-hydroxymethyl anthraquinone	270.24	3	5	1	0.07	High	medium_risk	*Tripterygium wilfordii* Hook.f
MID003059	13,14-Epoxide 9,11,12-hydroxytriptolide	378.42	3	7	1	0.85	High	low_risk	*Tripterygium wilfordii* Hook.f
MID004372	16-Hydroxy-19,20-epoxy-kaurane	304.47	1	2	0	1	High	low_risk	*Tripterygium wilfordii* Hook.f
MID004566	16-Hydroxytriptolide	376.4	2	7	2	0.85	High	low_risk	*Tripterygium wilfordii* Hook.f
MID004591	Hypolide methyl ether	326.43	0	3	2	0.57	High	medium_risk	*Tripterygium wilfordii* Hook.f
MID004875	Isoneotriptophenolide	342.43	1	4	2	0.57	High	low_risk	*Tripterygium wilfordii* Hook.f
MID005713	(+)-Medioresinol	388.41	2	7	5	0.43	High	medium_risk	*Tripterygium wilfordii* Hook.f
MID006737	Neotriptophenolide	342.43	1	4	2	0.57	High	low_risk	*Tripterygium wilfordii* Hook.f
MID008653	DL-Syringaresinol	418.44	2	8	6	0.45	High	low_risk	*Tripterygium wilfordii* Hook.f
MID009364	Triptonoterpene; 14-Hydroxy-abieta-8,11,13-trien-3-one	300.44	1	2	1	0.65	High	low_risk	*Tripterygium wilfordii* Hook.f
MID009365	Triptonoterpenol	346.46	2	4	3	0.67	High	low_risk	*Tripterygium wilfordii* Hook.f
MID009708	Wilforonide	220.26	0	3	0	0.69	High	low_risk	*Tripterygium wilfordii* Hook.f
MID009909	DIBP	278.34	0	4	8	0.5	High	low_risk	*Tripterygium wilfordii* Hook.f
MID010186	succinic acid	118.09	2	4	3	0.5	High	low_risk	*Tripterygium wilfordii* Hook.f
MID010204	syringaresinol	418.44	2	8	6	0.45	High	low_risk	*Tripterygium wilfordii* Hook.f
MID010234	(+)-Syringaresinol	418.44	2	8	6	0.45	High	low_risk	*Tripterygium wilfordii* Hook.f
MID010260	kaempferol	286.24	4	6	1	0	High	medium_risk	*Tripterygium wilfordii* Hook.f
MID010507	DBP	278.34	0	4	10	0.5	High	low_risk	*Tripterygium wilfordii* Hook.f
MID011265	protocatechualdehyde	138.12	2	3	1	0	High	low_risk	*Tripterygium wilfordii* Hook.f
MID011615	Cedar acid	198.17	2	5	3	0.22	High	low_risk	*Tripterygium wilfordii* Hook.f
MID011639	HX	136.11	2	3	0	0	High	medium_risk	*Tripterygium wilfordii* Hook.f
MID011860	40957-99-1	388.41	2	7	5	0.43	High	medium_risk	*Tripterygium wilfordii* Hook.f
MID012941	2,5-Dimethoxybenzoquinone	168.15	0	4	2	0.25	High	medium_risk	*Tripterygium wilfordii* Hook.f
MID012951	(+)-Medioresinol di-O-beta-D-glucopyranoside_qt	388.41	2	7	5	0.43	High	medium_risk	*Tripterygium wilfordii* Hook.f
MID012953	81827-74-9	342.43	1	4	2	0.57	High	low_risk	*Tripterygium wilfordii* Hook.f
MID012954	(1R,4aR,10aS)-5-hydroxy-1-(hydroxymethyl)-7-isopropyl-8-methoxy-1,4a-dimethyl-4,9,10,10a-tetrahydro-3H-phenthren-2-one	346.46	2	4	3	0.67	High	low_risk	*Tripterygium wilfordii* Hook.f
MID012956	triptolide	360.4	1	6	1	0.85	High	low_risk	*Tripterygium wilfordii* Hook.f
MID012957	Tripchlorolide	396.86	2	6	1	0.85	High	low_risk	*Tripterygium wilfordii* Hook.f
MID012960	Tripdiolide	376.4	2	7	1	0.85	High	low_risk	*Tripterygium wilfordii* Hook.f
MID012961	Triptonide	344.36	0	6	1	0.79	High	low_risk	*Tripterygium wilfordii* Hook.f
MID012965	Tryptophenolide	312.4	1	3	1	0.55	High	low_risk	*Tripterygium wilfordii* Hook.f
MID012968	5,8-Dihydroxy-7-(4-hydroxy-5-methyl-coumarin-3)-coumarin	352.29	3	7	1	0.05	High	medium_risk	*Tripterygium wilfordii* Hook.f
MID012969	HRP	220.22	4	4	3	0.18	High	medium_risk	*Tripterygium wilfordii* Hook.f
MID012972	8-Epilpganic acid_qt	214.22	3	5	1	0.7	High	low_risk	*Tripterygium wilfordii* Hook.f
MID012975	Canin	278.3	1	5	0	0.8	High	low_risk	*Tripterygium wilfordii* Hook.f
MID012977	Celafurine	369.46	2	4	3	0.43	High	medium_risk	*Tripterygium wilfordii* Hook.f
MID012983	Dunnisinin	226.23	1	5	2	0.73	High	low_risk	*Tripterygium wilfordii* Hook.f
MID012985	trans-Nepetalactone	166.22	0	2	0	0.7	High	low_risk	*Tripterygium wilfordii* Hook.f
MID012986	Isoxanthohumol	354.4	2	5	4	0.29	High	medium_risk	*Tripterygium wilfordii* Hook.f
MID012987	Neouralenol	370.35	5	7	3	0.15	High	medium_risk	*Tripterygium wilfordii* Hook.f
	[(3aR,4S,6E,9S,10Z,11aR)-9-hydroxy-6,10-dimethyl-3-methylene-2-oxo-3a,4,5,8,9,11a-hexahydrocyclodeca [b]furan-4-yl] (E)-2-methylbut-2-enoate	346.42	1	5	3	0.5	High	medium_risk	*Tripterygium wilfordii* Hook.f
MID012992	Tripdiotolnide	360.4	2	6	1	0.7	High	low_risk	*Tripterygium wilfordii* Hook.f
MID012993	Hypodiolide A	318.45	1	3	0	0.95	High	low_risk	*Tripterygium wilfordii* Hook.f
MID012996	Norathyriol	260.2	4	6	0	0	High	low_risk	*Tripterygium wilfordii* Hook.f
MID012997	Triptinin B	314.42	2	3	2	0.55	High	low_risk	*Tripterygium wilfordii* Hook.f
MID012999	Triptoditerpenic acid B	328.45	1	3	3	0.57	High	low_risk	*Tripterygium wilfordii* Hook.f
MID013005	(3E,7E)-2alpha,10beta,13alpha-Triacetoxy-5alpha,20-dihydroxy-3,8-seco-taxa-3,7,11-trien-9-one	492.56	2	9	7	0.62	High	ambiguous	*Tripterygium wilfordii* Hook.f
MID013011	Triptolidenol	376.4	2	7	1	0.85	High	low_risk	*Tripterygium wilfordii* Hook.f
MID013012	Triptonide	358.39	0	6	1	0.8	High	low_risk	*Tripterygium wilfordii* Hook.f
MID013013	Triptonoditerpenic acid	344.44	2	4	3	0.57	High	low_risk	*Tripterygium wilfordii* Hook.f
MID013014	11-Hydroxy-14,15alpha-epoxytabersonine	368.43	2	5	3	0.57	High	low_risk	*Tripterygium wilfordii* Hook.f
MID013015	Triptonoterpene methyl ether	330.46	1	3	2	0.67	High	low_risk	*Tripterygium wilfordii* Hook.f
MID013016	Triptonoterpene	300.44	1	2	1	0.65	High	low_risk	*Tripterygium wilfordii* Hook.f
MID013031	Wilfordic acid	223.23	2	5	5	0.36	High	low_risk	*Tripterygium wilfordii* Hook.f
MID013037	104331-87-5	220.26	0	3	0	0.69	High	low_risk	*Tripterygium wilfordii* Hook.f
MID013040	Wilsonine	343.42	0	5	3	0.6	High	low_risk	*Tripterygium wilfordii* Hook.f
MID013047	99694-86-7	376.4	2	7	1	0.85	High	low_risk	*Tripterygium wilfordii* Hook.f
MID013048	TRIPTONOLIDE	326.39	1	4	1	0.5	High	low_risk	*Tripterygium wilfordii* Hook.f
MID013051	(2R,3R,4S)-4-(4-hydroxy-3-methoxy-phenyl)-7-methoxy-2,3-dimethylol-tetralin-6-ol	360.4	4	6	5	0.4	High	medium_risk	*Tripterygium wilfordii* Hook.f
MID013722	caffeine	194.19	0	3	0	0.38	High	medium_risk	*Tripterygium wilfordii* Hook.f
MID014181	Zhebeiresinol	280.27	1	6	3	0.5	High	low_risk	*Tripterygium wilfordii* Hook.f
MID014393	fraxetin	208.17	2	5	1	0.1	High	low_risk	*Tripterygium wilfordii* Hook.f
MID016058	(2R,3R,4S)-4-(4-hydroxy-3,5-dimethoxy-phenyl)-5,7-dimethoxy-2,3-dimethylol-tetralin-6-ol	420.45	4	8	7	0.45	High	low_risk	*Tripterygium wilfordii* Hook.f
MID016077	4-[(1R,3aS,4R,6aS)-4-(4-hydroxy-3,5-dimethoxyphenyl)-1,3,3a,4,6,6a-hexahydrofuro [4,3-c]furan-1-yl]-2,6-dimethoxyphenol	418.44	2	8	6	0.45	High	low_risk	*Tripterygium wilfordii* Hook.f
MID017234	3-hydroxy-1-(3,5-dimethoxy-4-hydroxyphenyl)propan-1-one	226.23	2	5	5	0.36	High	low_risk	*Tripterygium wilfordii* Hook.f
MID018996	3,3′-bis-(3,4-dihydro-4-hydroxy-6-methoxy)-2H-1-benzopyran	358.39	2	6	3	0.4	High	medium_risk	*Tripterygium wilfordii* Hook.f
MID022242	Antiarol	184.19	1	4	3	0.33	High	low_risk	*Tripterygium wilfordii* Hook.f
MID004896	Isopinocamphone	152.23	0	1	0	0.9	High	low_risk	*Lysimachia christinae* Hance
MID005770	Menthol	156.27	1	1	1	1	High	low_risk	*Lysimachia christinae* Hance
MID005771	Menthol-b	156.27	1	1	1	1	High	low_risk	*Lysimachia christinae* Hance
MID007428	L-Pinocamphone	152.23	0	1	0	0.9	High	low_risk	*Lysimachia christinae* Hance
MID009860	apigenin	270.24	3	5	1	0	High	medium_risk	*Lysimachia christinae* Hance
MID009925	ent-Epicatechin	290.27	5	6	1	0.2	High	medium_risk	*Lysimachia christinae* Hance
MID009949	quercetin	302.24	5	7	1	0	High	medium_risk	*Lysimachia christinae* Hance
MID009953	PHB	138.12	2	3	1	0	High	low_risk	*Lysimachia christinae* Hance
MID009966	Nol	142.24	0	1	7	0.89	High	low_risk	*Lysimachia christinae* Hance
MID009968	(L)-alpha-Terpineol	154.25	1	1	1	0.8	High	low_risk	*Lysimachia christinae* Hance
MID009980	CAM	152.23	0	1	0	0.9	High	low_risk	*Lysimachia christinae* Hance
MID010041	(R)-lilool	154.25	1	1	4	0.6	High	low_risk	*Lysimachia christinae* Hance
MID010093	Rhamnocitrin	300.26	3	6	2	0.06	High	medium_risk	*Lysimachia christinae* Hance
MID010193	isorhamnetin	316.26	4	7	2	0.06	High	medium_risk	*Lysimachia christinae* Hance
MID010260	kaempferol	286.24	4	6	1	0	High	medium_risk	*Lysimachia christinae* Hance
MID010435	O-Methylthymol	164.24	0	1	2	0.45	High	medium_risk	*Lysimachia christinae* Hance
MID010526	patchouli alcohol	222.37	1	1	0	1	High	low_risk	*Lysimachia christinae* Hance
MID010546	l-Menthone	154.25	0	1	1	0.9	High	low_risk	*Lysimachia christinae* Hance
MID010700	Cedrol	222.37	1	1	0	1	High	low_risk	*Lysimachia christinae* Hance
MID011127	o-Acetyl-p-cresol	150.17	1	2	1	0.22	High	low_risk	*Lysimachia christinae* Hance
MID011208	49070_FLUKA	222.37	1	1	0	1	High	low_risk	*Lysimachia christinae* Hance
MID011498	acacetin	284.26	2	5	2	0.06	High	medium_risk	*Lysimachia christinae* Hance
MID011502	Perillyl alcohol	152.23	1	1	2	0.6	High	low_risk	*Lysimachia christinae* Hance
MID011777	Pulegone	152.23	0	1	0	0.7	High	low_risk	*Lysimachia christinae* Hance
MID011806	(-)-Caryophyllene oxide	220.35	0	1	0	0.87	High	medium_risk	*Lysimachia christinae* Hance
MID011844	thymol	150.22	1	1	1	0.4	High	low_risk	*Lysimachia christinae* Hance
MID012135	Hesperetin	302.28	3	6	2	0.19	High	medium_risk	*Lysimachia christinae* Hance
MID012156	beta-Ionone	192.3	0	1	2	0.62	High	low_risk	*Lysimachia christinae* Hance
MID017581	2-Hexanoylfuran	166.22	0	2	5	0.5	High	low_risk	*Lysimachia christinae* Hance

### 3.3 C-T network construction and analysis

**FIGURE 3:**
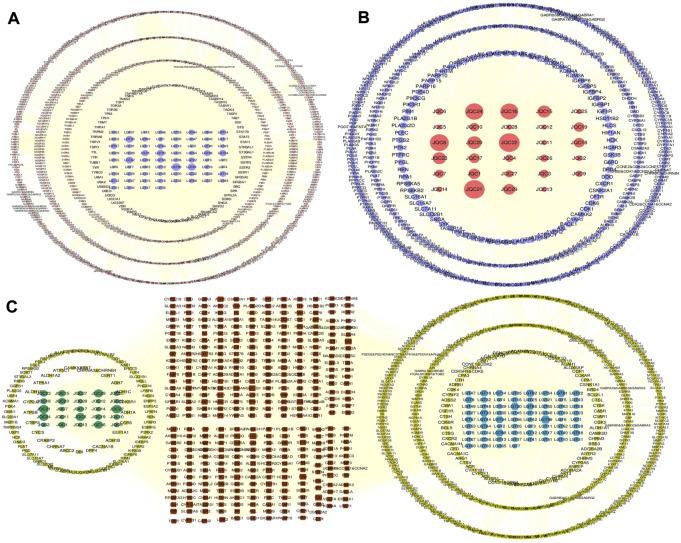
The C-T network of LGT and JQC. **(A)** C-T network of LGT. **(B)** C-T network of JQC. **(C)** Common and specific C-T network for LGT and JQC. The blue nodes represent components of LGT. The green nodes represent components of JQC. The red nodes means common targets of LGT and JCQ, and the yellow ones as the specific targets.

To facilitate analysis of the complex relationship between the active components and their targets in LGT and JQC, a component-target (C-T) network was constructed by using Cytoscape3.9.1 (Figure 3). The results showed that in the common C-T network of LGT-JQC, active components of LGT and JQC are related to multiple targets, resulting in 5778 associations between 97 active components and 944 targets. The average number of targets for per component is 11.2, and the average number of components for per target is 8.1, which reflect the characteristics of multi-component and multi-target mediated synergistic reaction and the complexity of the action mechanism of TCM. The most targeted components were (2R,3R, 4S)-4-(4-hydroxy-3-methoxyphenyl)-7-methoxy-2,3-dimethyl-tetralin-6-ol (LGT59, degree = 168), followed by quercetin (JQC22, degree = 159), poly-β-hydroxybutyric acid (JQC21, degree = 158) and zhebeisu (LGT61, degree = 152). These results indicate these shared components may play synergetic effect in the combination treatment of lung cancer.

There were 525 shared targets in LGT and JQC (Figure 2B), and among these common targets, aromatase (CYP19A1, degree = 64) was targeted by the most components in LGT and JQC. Previous reports have confirmed that aromatase had biological activity in lung cancer cells, and the polymorphisms of CYP19A1 may be related to the increased risk of lung cancer (Zhang et al., 2013). In addition, among common targets of LGT and JQC, a series of carbonic anhydrase such as CA12, CA2, CA1, and CA9 are also highly expressed, and published evidence shown that carbonic anhydrase can affect the proliferation of non-small cell lung cancer (NSCLC) through the Wnt/β-catenin signaling pathway (Wang et al., 2020a).

In LGT, there were 4120 interactions between 68 active components and 901 targets. Except for common targets, many targets of components in LGT are directly or indirectly related to lung cancer. Such as PTPN1 (degree = 44) and AKT1 (degree = 42). PTPN1 (degree = 44), a unique target of LGT has been proved to promote the proliferation and metastasis of NSCLC and high expression in lung cancer tissues (Wang et al., 2013). GPR37 has attracted much attention as a therapeutic target for lung cancer. The down-regulation of GPR37 expression can significantly inhibit the proliferation and migration of lung adenocarcinoma *in vitro* and *in vivo* (Xie et al., 2022). These results indicate that the high degree genes specifically targeted by LGT are mostly related to the pathogenic mechanism of lung cancer.

In JQC, there were 1656 interactions between the 29 active components and 514 targets. In addition to the significant targets already discussed above among the common targets, we can also focus on several other targets that are significantly associated with lung cancer. For example, ESR2 (degree = 13), also known as ERβ, it could promote lung cancer invasion *via* increasing CXCR4 expression (Liu et al., 2022). In addition, the TTR (degree = 13) is selectively highly expressed in lung cancer cells and can be secreted out of cells (Hao et al., 2016).

In summary, these results suggested that the active ingredients between LGT and JQC may treat lung cancer through multi-target synergy, and achieve the effects of promoting efficiency and reducing toxicity after compatibility.

### 3.4 Analysis of pathogenic genes of lung cancer

**FIGURE 4:**
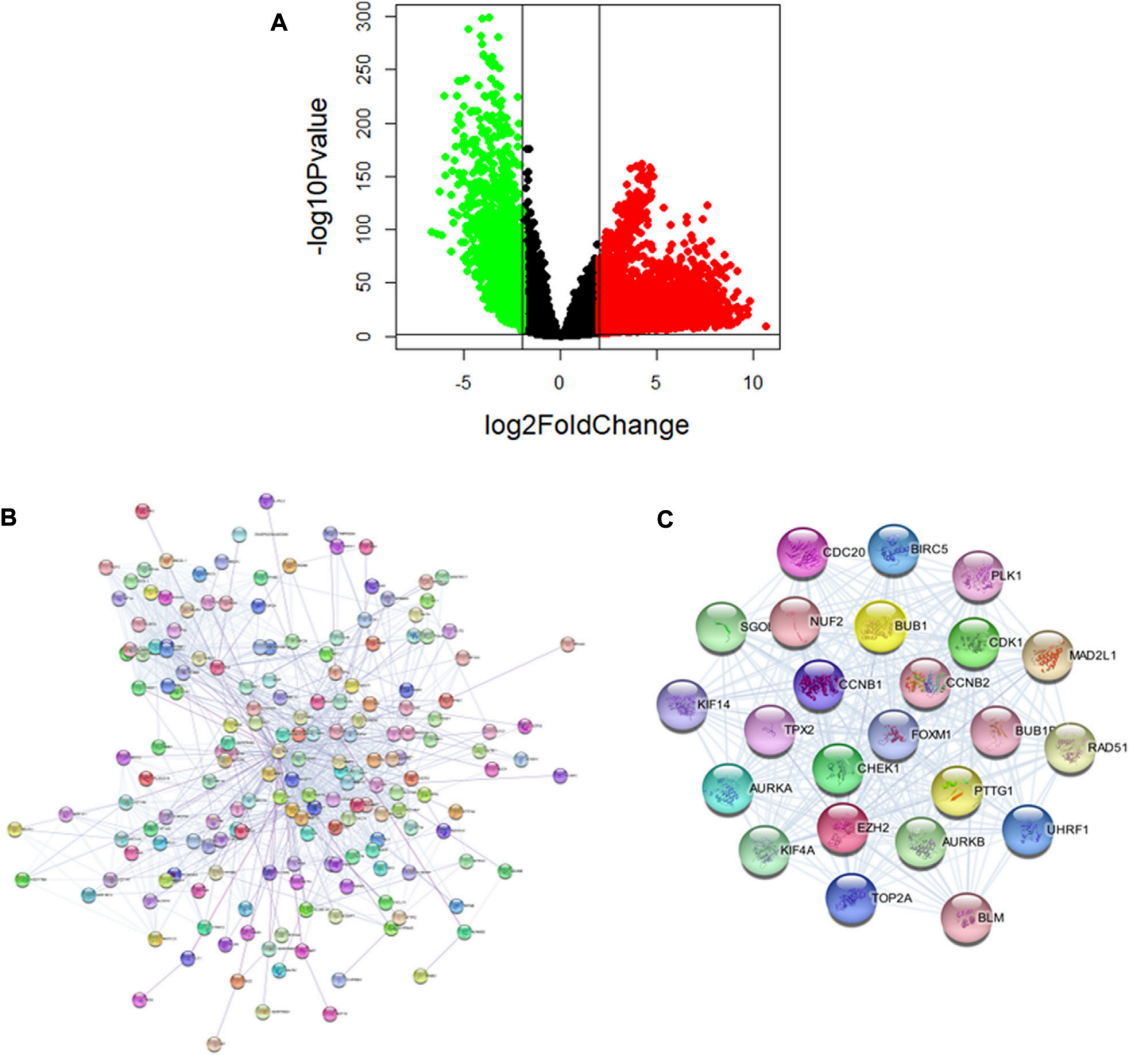
Analysis of pathogenic genes of Lung cancer. **(A)** Significant DEGs of lung cancer. **(B)** PPI network of lung cancer pathogenic genes. **(C)** The sub-networks were identified by Cytoscape MCODE plugin.

To determine the pathogenic genes in lung cancer, we downloaded the data of lung cancer from TCGA, and obtained 8319 differential expression genes (DEGs), of which 5867 genes were up-regulated and 2452 genes were down-regulated (Figure 4A). Next, we identified the key pathogenic genes using the PPI network of DEGs. The PPI network of DEGs was constructed using the STRING (https://cn.string-db.org/) and Cytoscape (Figure 4B). Modular analysis of PPI networks was performed by using the MCODE plugin (degree cutoff = 2, node score cutoff = 0.2, k-core = 2, and max. depth = 100). We screened the top cluster with the highest clustering score (score = 22.957) to do further analyses (Figure 4C). Then, the central node gene (more than 10 connections/interactions) was figured out, and the top ranked 24 highly linked genes were RAD51, CCNB1, CCNB2, KIF14, EZH2, CDC20, FoxM1, AURKA, BUB1B, SGOL1, etc. The functions of these genes in this module are mainly related to nuclear division, organelle fission, mitotic nuclear division, sister chromatid segregation, Mitotic Cell Cycle Phase Transition, and chromosome segregation. EZH2 is known to play an important role in the occurrence, development and metastasis of cancer, while studies have demonstrated that many lncRNA promote the function of lung cancer cells by interacting with EZH2 to silence tumor suppressor factors (Su et al., 2018). In addition, CDK1 mRNA expression was negatively correlated with the overall survival of lung cancer patients, and CDK1/Sox2 regulated the activity of liver cancer stem cells in a positively regulated manner (Huang et al., 2021). Other studies have demonstrated that CCNB1 participates in the lung cancer related lncRNA-miRNA-mRNA ceRNA network (Wu et al., 2020). AURKA is an important gene for cell cycle regulation. CBLC could delay the accumulation and activation of AURKA through consumption to prevent cancer cells from entering cell division and increase the apoptosis of lung cancer cells (Hong et al., 2022). In addition, FoxM1 as a tumor marker for lung cancer has been commonly recognized (Gao et al., 2021). In summary, these results have revealed a complex network of multiple targets and multiple mechanisms among lung cancer -causing genes.

### 3.5 Identification of toxic targets

Literature retrieval and statistics showed that the most serious adverse reactions of LGT were mainly in the bone marrow and blood system, followed by the digestive system and renal system [Supplementary Table S2]. These adverse reactions involve many target genes. We defined the toxicity targets that are related to adverse reactions in multiple systems and the number of literature reports is greater than 10. According to the above criteria, the top 10 important toxicity targets are: TP53, CFTR, AGT, ESR1, PPARG, CDKN2A, MDM2, AR, JAK2, and AGTR1. As an important tumor suppressor gene, TP53 is associated with apoptosis and cell cycle regulation. At the same time, it is a disease target in the adverse reactions of LGT—palpitation, pancytopenia, renal failure, acute renal insufficiency, and anemia. The frequency of TP53 mutation increased with the progression of multiple myeloma (Jovanovic et al., 2018). In addition, TP53 regulated the invasion of renal clear cell carcinoma through PI3K/PTEN/AKT signal (Lei, 2020). All these indicated that TP53 was an important toxic target of LGT. The toxicity of LGT in cardiovascular system often leads to the occurrence of palpitation, arrhythmia and bradycardia (Hua et al., 2011), and in the bone marrow and blood system often lead to anemia and pancytopenia (LiuMiaoHui and Yumeng, 2014). AGT, as an essential component of the renin-angiotensin system, is an effective blood pressure regulator and an important target for diseases in the cardiovascular system, bone marrow and blood system. It was closely related to the adverse reactions in the cardiovascular system and bone marrow blood system caused by the toxicity of LGT. PPARG is expressed to different degrees in adipose tissues, liver, skeletal muscle, kidney, pancreas and other tissues, and is also one of the pathogenic targets of lung cancer. More importantly, PPARG is a disease-related target in the development of LGT adverse reactions such as renal failure, infrequent menstruation, neutropenia, hepatomegaly, and other reproductive and hepatic toxicity. Taken together, these results reveal a complex network of multiple systems and multiple targets between the toxicities and side effects of LGT.

### 3.6 Construction and validation of quantitative efficacy-toxicity network of LGT

#### 3.6.1 Construction of quantitative efficacy-toxicity network of LGT

**FIGURE 5:**
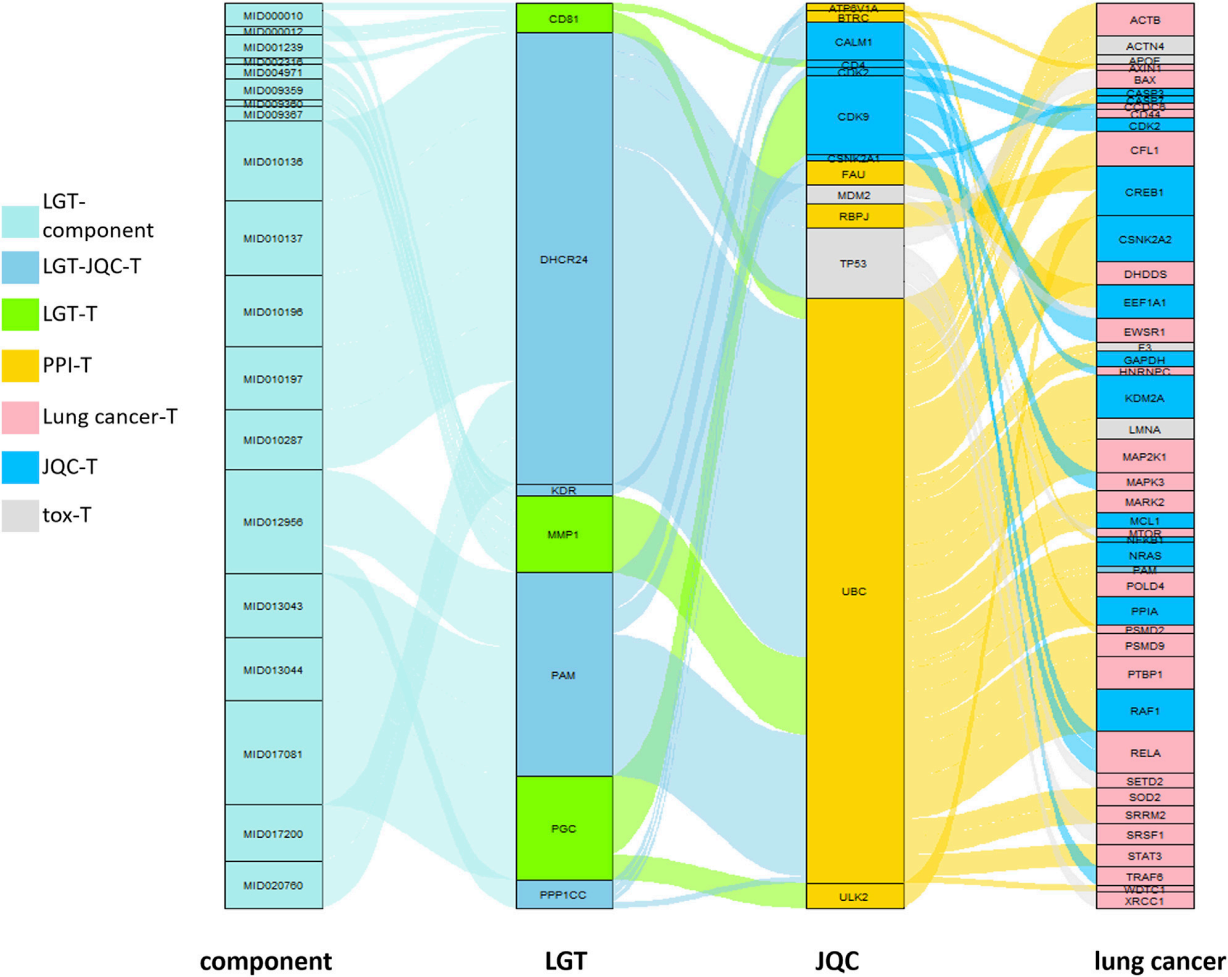
quantitative efficacy-toxicity network of LGT.

To better detect the gradual transfer of LGT from target to pathogenic gene during treatment, we first need to obtain the initial influence coefficients of components on the target. To solve this problem, we design a matrix decomposition model with the interaction of components and targets as well as the expression of target genes as inputs, finally we obtain a total of 10,423 component-target regulations with initial influence coefficients, based on the initial impact coefficient, we designed a new propagation model. The propagation model not only considered that the initial influence coefficient would transmit the effect to other effective targets but also to other targets related to side effects in the propagation process, so the model could reflect the cascade effect mechanism of drugs *in vivo* in a quantitative manner. Through this model we finally obtained 1,396,561 GTCs [Supplementary Table S3]. These GTCs are complex and large. How to quickly extract important information from many GTCs is a key step in deciphering the potential molecular mechanism of the compatibility of LGT and JQC in the treatment of lung cancer. Here, the GTCs with high scores are considered to have high correlation with LGT combined with JQC in the treatment of lung cancer. Therefore, the median of the final scores of all GTCs is selected as the screening criterion, and GTCs with scores higher than the median are retained and defined as efficacy-toxicity network for further analysis. We found that most of the first 50 GTCs with the highest scores passed through both LGT and JQC targets, suggesting that LGT and JQC had synergistic effects in the treatment of lung cancer (Figure 5). Detail of the GTCs in the efficacy-toxicity network are shown in Table 3.

**TABLE 3:** 50 representative GTFs.

inital_value	Gene transmission chain	Spread value	Loss value	tox_begin value	tox_fin value	JQC_begin value	JQC_fin value	fin_value
86.93	DHCR24 [JQC]-UBC-CFL1	26.74	60.19	0.00	0.00	86.93	63.29	90.03
86.93	DHCR24 [JQC]-UBC-MAP2K1	25.54	61.39	0.00	0.00	86.93	60.45	85.99
86.93	DHCR24 [JQC]-UBC-ACTB	25.29	61.64	0.00	0.00	86.93	59.86	85.15
86.93	DHCR24 [JQC]-UBC-PTBP1	25.03	61.90	0.00	0.00	86.93	59.25	84.28
86.93	DHCR24 [JQC]-UBC-CSNK2A2 [JQC]	23.06	63.87	0.00	0.00	86.93	54.58	77.65
86.93	DHCR24 [JQC]-UBC-PPIA [JQC]	22.44	64.48	0.00	0.00	86.93	53.12	75.57
86.93	DHCR24 [JQC]-UBC-KDM2A [JQC]	22.18	64.75	0.00	0.00	86.93	52.50	74.68
86.93	DHCR24 [JQC]-UBC-RAF1 [JQC]	21.59	65.34	0.00	0.00	86.93	51.10	72.69
270.12	PGC-ULK2-CREB1 [JQC]	33.41	236.71	0.00	0.00	33.41	33.41	66.83
86.93	DHCR24 [JQC]-UBC-NRAS [JQC]	19.12	67.81	0.00	0.00	86.93	45.25	64.36
86.93	DHCR24 [JQC]-RBPJ-CREB1 [JQC]	13.75	73.18	0.00	0.00	86.93	50.52	64.27
65.71	PAM [JQC]-UBC-PSMD9	19.13	46.58	0.00	0.00	65.71	44.33	63.46
65.71	PAM [JQC]-UBC-POLD4	18.81	46.90	0.00	0.00	65.71	43.60	62.42
65.71	PAM [JQC]-FAU-EEF1A1 [JQC]	15.18	50.53	0.00	0.00	65.71	47.17	62.36
65.71	PAM [JQC]-UBC-DHDDS	18.61	47.10	0.00	0.00	65.71	43.13	61.74
270.12	PGC-CDK9 [JQC]-EWSR1	30.79	239.33	0.00	0.00	40.06	30.79	61.58
270.12	PGC-CDK9 [JQC]-RELA	30.27	239.85	0.00	0.00	40.06	30.27	60.54
65.71	PAM [JQC]-UBC-MARK2	17.56	48.15	0.00	0.00	65.71	40.70	58.27
65.71	PAM [JQC]-UBC-STAT3	17.51	48.20	0.00	0.00	65.71	40.58	58.10
86.93	DHCR24 [JQC]-UBC-LMNA [TOX]	23.37	63.55	23.37	23.37	86.93	55.32	55.32
86.93	DHCR24 [JQC]-TP53 [TOX]-SRSF1	16.94	69.98	27.22	16.94	86.93	54.11	54.11
86.93	DHCR24 [JQC]-UBC-ACTN4 [TOX]	21.66	65.27	21.66	21.66	86.93	51.26	51.26
65.71	PAM [JQC]-CALM1 [JQC]-RELA	13.53	52.18	0.00	0.00	65.71	36.22	49.75
65.71	PAM [JQC]-CALM1 [JQC]-TRAF6	13.52	52.19	0.00	0.00	65.71	36.21	49.73
86.93	DHCR24 [JQC]-UBC-SRRM2	14.60	72.32	0.00	0.00	86.93	34.57	49.17
86.93	DHCR24 [JQC]-TP53 [TOX]-BAX	15.03	71.90	27.22	15.03	86.93	48.01	48.01
270.12	PGC-CDK9 [JQC]-MAPK3	23.90	246.22	0.00	0.00	40.06	23.90	47.80
65.71	PAM [JQC]-UBC-SOD2	13.54	52.17	0.00	0.00	65.71	31.38	44.93
86.93	DHCR24 [JQC]-TP53 [TOX]-XRCC1	14.04	72.89	27.22	14.04	86.93	44.84	44.84
272.62	MMP1-UBC-CSNK2A2 [JQC]	20.97	251.65	0.00	0.00	20.97	20.97	41.94
272.62	MMP1-UBC-MCL1 [JQC]	20.84	251.78	0.00	0.00	20.84	20.84	41.68
272.62	MMP1-UBC-KDM2A [JQC]	20.17	252.45	0.00	0.00	20.17	20.17	40.33
272.62	MMP1-UBC-GAPDH [JQC]	20.12	252.50	0.00	0.00	20.12	20.12	40.23
272.62	MMP1-UBC-RAF1 [JQC]	19.63	252.98	0.00	0.00	19.63	19.63	39.26
86.93	DHCR24 [JQC]-TP53 [TOX]-SETD2	12.18	74.75	27.22	12.18	86.93	38.88	38.88
270.12	PGC-CDK9 [JQC]-CDK2 [JQC]	19.14	250.98	0.00	0.00	40.06	19.14	38.27
86.93	DHCR24 [JQC]-MDM2 [TOX][JQC]-EEF1A1 [JQC]	3.71	83.22	11.75	3.71	86.93	27.42	27.42
86.93	DHCR24 [JQC]-UBC-APOE [TOX]	10.46	76.47	10.46	10.46	86.93	24.75	24.75
65.71	PAM [JQC]-UBC-F3 [TOX][JQC]	10.50	55.21	10.50	10.50	65.71	24.33	24.33
18.84	PPP1CC [JQC]-CDK2 [JQC]-HNRNPC	9.06	9.78	0.00	0.00	18.84	12.78	21.84
86.93	DHCR24 [JQC]-MDM2 [TOX][JQC]-MTOR	2.93	84.00	11.75	2.93	86.93	21.70	21.70
18.84	PPP1CC [JQC]-ATP6V1A-PSMD2	8.14	10.71	0.00	0.00	18.84	12.28	20.42
39.05	CD81-UBC-CASP3 [JQC]	10.15	28.90	0.00	0.00	10.15	10.15	20.30
39.05	CD81^−^CD4 [JQC]-CD44	10.12	28.93	0.00	0.00	19.04	10.12	20.23
39.05	CD81-UBC-CASP7 [JQC]	10.09	28.96	0.00	0.00	10.09	10.09	20.17
39.05	CD81-UBC-PAM [JQC]	8.10	30.95	0.00	0.00	8.10	8.10	16.20
18.84	PPP1CC [JQC]-CSNK2A1 [JQC]-CCDC6	6.53	12.31	0.00	0.00	18.84	9.60	16.14
18.84	PPP1CC [JQC]-UBC-WDTC1	6.00	12.85	0.00	0.00	18.84	10.14	16.14
18.77	KDR [JQC]-BTRC-NFKB1 [JQC]	3.62	15.15	0.00	0.00	18.77	11.28	14.90
18.77	KDR [JQC]-BTRC-AXIN1	3.61	15.16	0.00	0.00	18.77	11.24	14.85

#### 3.6.2 Validation of quantitative efficacy-toxicity network for LGT

In order to verify whether the constructed quantitative efficacy-toxicity network of LGT better reveals the mechanism of the combination of LGT and JQC in the treatment of lung cancer, we adopted three strategies to verify the accuracy, reliability and effectiveness of the quantitative efficacy-toxicity network of LGT. The first strategy is to observe the percentage of the number of component targets with high initial influence coefficient obtained by matrix decomposition method to all component targets of LGT. Targets with a higher initial coefficient of influence were considered to be significant targets for LGT (LGT-ST), and coverage was defined as the number of LGT-ST as a percentage of the number of targets for all components of LGT and the lung cancer pathogenic gene. The high coverage indicated that LGT-ST could retain most of the effective targets. The second strategy aims to observe and compare the number of targets for LGT and lung cancer in the screened GTSs with the number of targets for all components of LGT and pathogenic genes for lung cancer. High coverage may indicate that the GTCs retains the majority of LGT and lung cancer targets. The third strategy was mainly used to observe whether the gene enrichment pathways in the quantitative efficacy-toxicity network covered as much as possible of the gene enrichment pathways for LGT and lung cancer.

#### 3.6.3 Verify the coverage of the LGT-ST

**FIGURE 6:**
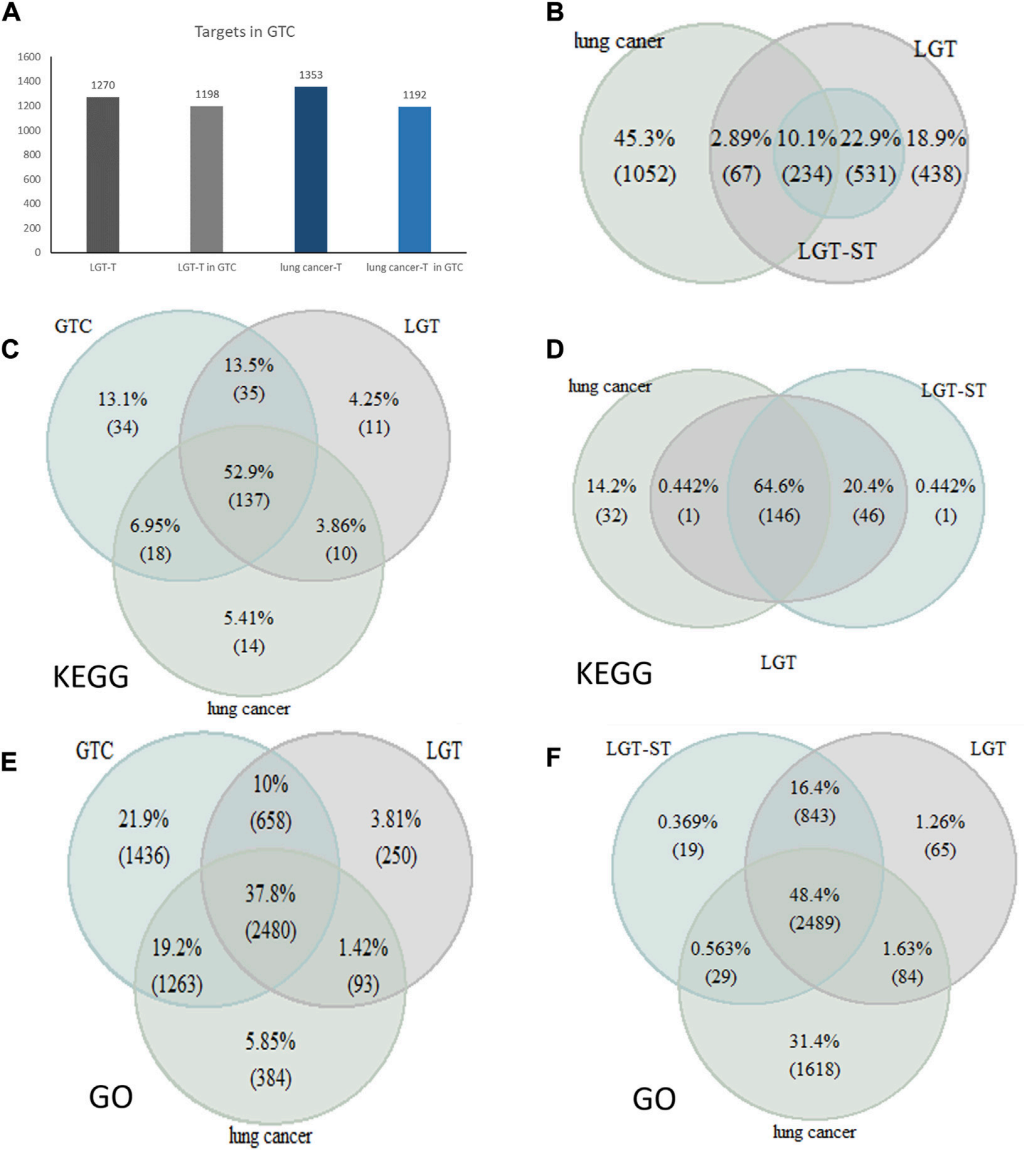
Validation of quantitative efficacy-toxicity network of LGT. **(A)** The number of LGT targets and lung cancer targets in the GTC; **(B)** Number of overlapping genes of LGT, Lung cancer and LGT-ST; **(C,D)**: Coverage of pathway analysis between LGT targets and lung cancer targets with GTC **(C)** and LGT-ST **(D)**; **(E,F)**: Coverage of GO function enrichment between LGT targets and lung cancer targets with GTC **(E)** and LGT-ST **(F)**.

We collected the lung cancer pathogenic genes reported in the published literature and database (Supplementary Table S4). We found that there are 765 LGT-ST genes in total, and among the 301 target genes shared by LGT and lung cancer, the coverage rate of LGT-ST is 77.7%. This confirmed that the LGT-ST had a high degree of consistency with the effective targets of LGT (Figure 6), and there was a high coverage rate between the common targets of LGT and lung cancer, which also indicated that the LGT-ST obtained by matrix decomposition could accurately reveal the influence coefficient between LGT components and targets. Therefore, the propagation model in which the initial influence coefficient participated in the calculation was more reliable.

#### 3.6.4 Verifying target coverage in the GTC

The number of LGT targets and lung cancer targets in the GTCs was counted and compared with the number collected through literature retrieval. Fortunately, 1198 LGT targets and 1192 lung cancer targets were included in the GTCs, accounting for 94% and 88% of all LGT targets and lung cancer targets (Figure 5A). This result confirmed that the GTCs obtained by the transmission model had a high degree of coincidence with LGT targets and lung cancer targets.

#### 3.6.5 Validated the genes enriched Pathways in efficacy-toxicity network

We obtained the gene enrichment pathways for the efficacy-toxicity network、LGT and lung cancer from KEGG (Kanehisa and Goto, 2000). The analysis results showed that the GO functional enrichment of LGT-ST accounted for 95.7% and 74.49% of gene GO functional enrichment in LGT and lung cancer, respectively. The functional enrichment of GO by targets in the GTC accounted for 90.1% and 88.6% of the functional enrichment of LGT and lung cancer, respectively. In addition, 85.42% of pathways in the genes enriched pathways of LGT-ST were the same as those for LGT and lung cancer. Genes enriched pathways of targets in the GTCs accounted for 89.1% and 86.6% of the pathways for LGT and lung cancer, respectively (Figures 5C–E). This result demonstrated high coincidence of the GTCs with LGT and lung cancer at the gene level, and also indicated that the quantitative efficacy-toxicity network of LGT constructed by us can maximize the conservation of the functional pathways of LGT and JQC in the treatment of lung cancer.

### 3.7 Potential mechanism analysis of the combination of LGT and JQC in the treatment of lung cancer

**FIGURE 7:**
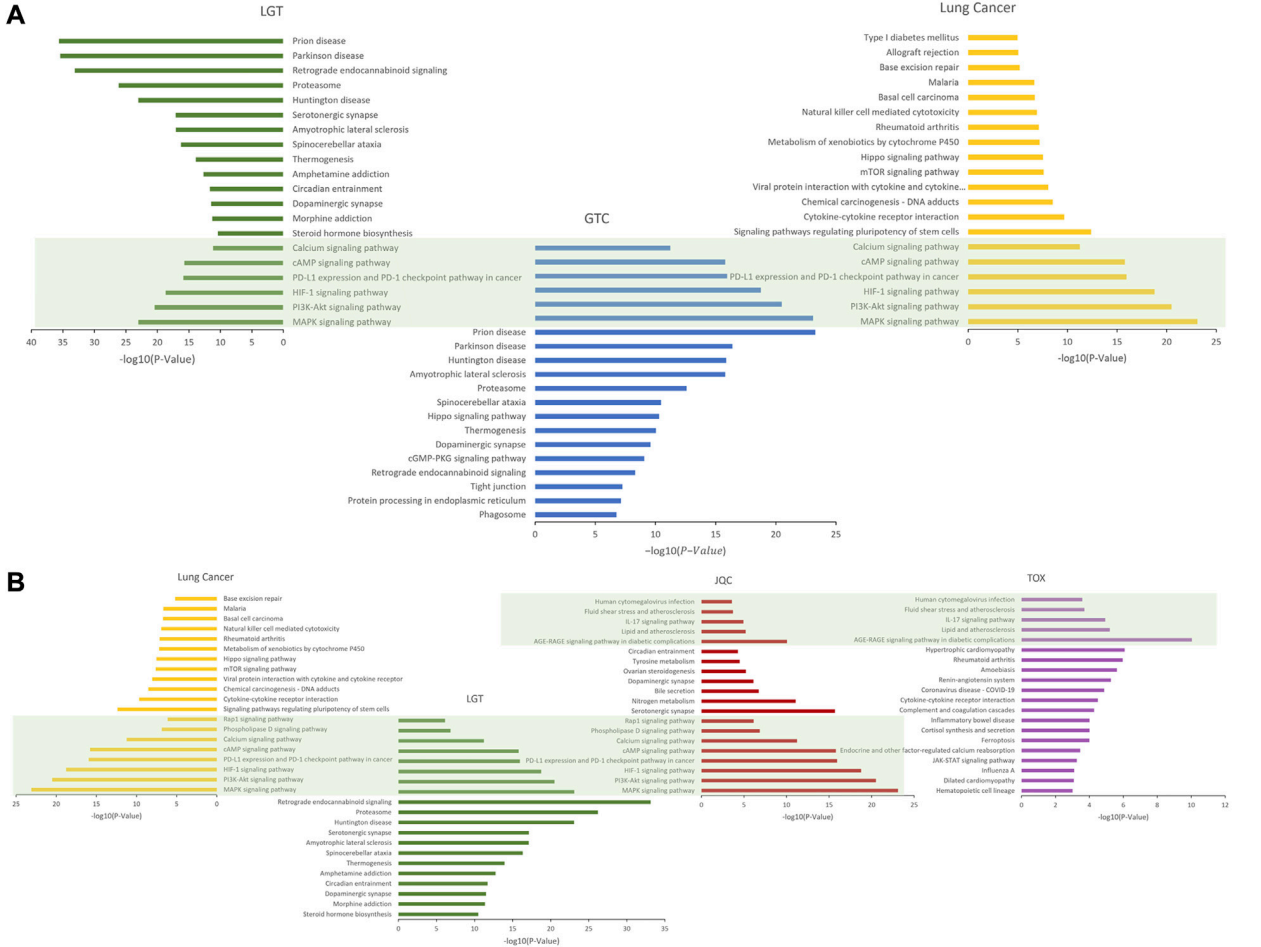
The enrichment pathway map of LGT and JQC in the treatment of lung cancer. **(A)** Target enrichment pathways of LGT, LUAD and GTC; **(B)** Gene enrichment pathways for toxic targets of LGT, LUAD, JQC, and LGT.

To reveal the potential mechanism of the compatibility of LGT and JQC in the treatment of lung cancer, we conducted path enrichment analysis on the toxic targets of LGT, JQC, lung cancer, and LGT as well as related genes in the GTC. First, genes of LGT were enriched in 194 pathways, genes of lung cancer were enriched in 180 pathways, and related genes in GTC were enriched in 225 pathways, among which there were 137 common pathways, the more significant ones were MAPK signaling pathway, PI3K-Akt signaling pathway, HIF-1 signaling pathway, cAMP signaling pathway, etc. In JQC, genes were enriched along 145 pathways, while toxicity targets for LGT were enriched along 69 pathways. We performed unified analysis on the obtained pathways of LGT, JQC, lung cancer and LGT toxicity targets, and found that the common pathway of LGT, JQC and lung cancer covered 114 pathways. These include: MAPK signaling pathway, PI3K-Akt signaling pathway, cAMP signaling pathway, PD-L1 expression and PD-1 checkpoint pathway in cancer, Rap1 signaling pathway, etc. JQC pathways covered 42 pathways of LGT toxicity targets, including IL-17 signaling pathway, Fluid Sheet Stress and Atherosclerosis, and Human cytomegalovirus infection. These results indicate that LGT and JQC can exert the effect of treating lung cancer through multiple common pathways, and JQC also has multiple common pathways with the toxic targets of LGT (Figure 7), which may be the key to the mechanism of JQC in the promoting efficiency and reducing toxicity of LGT.

**FIGURE 8:**
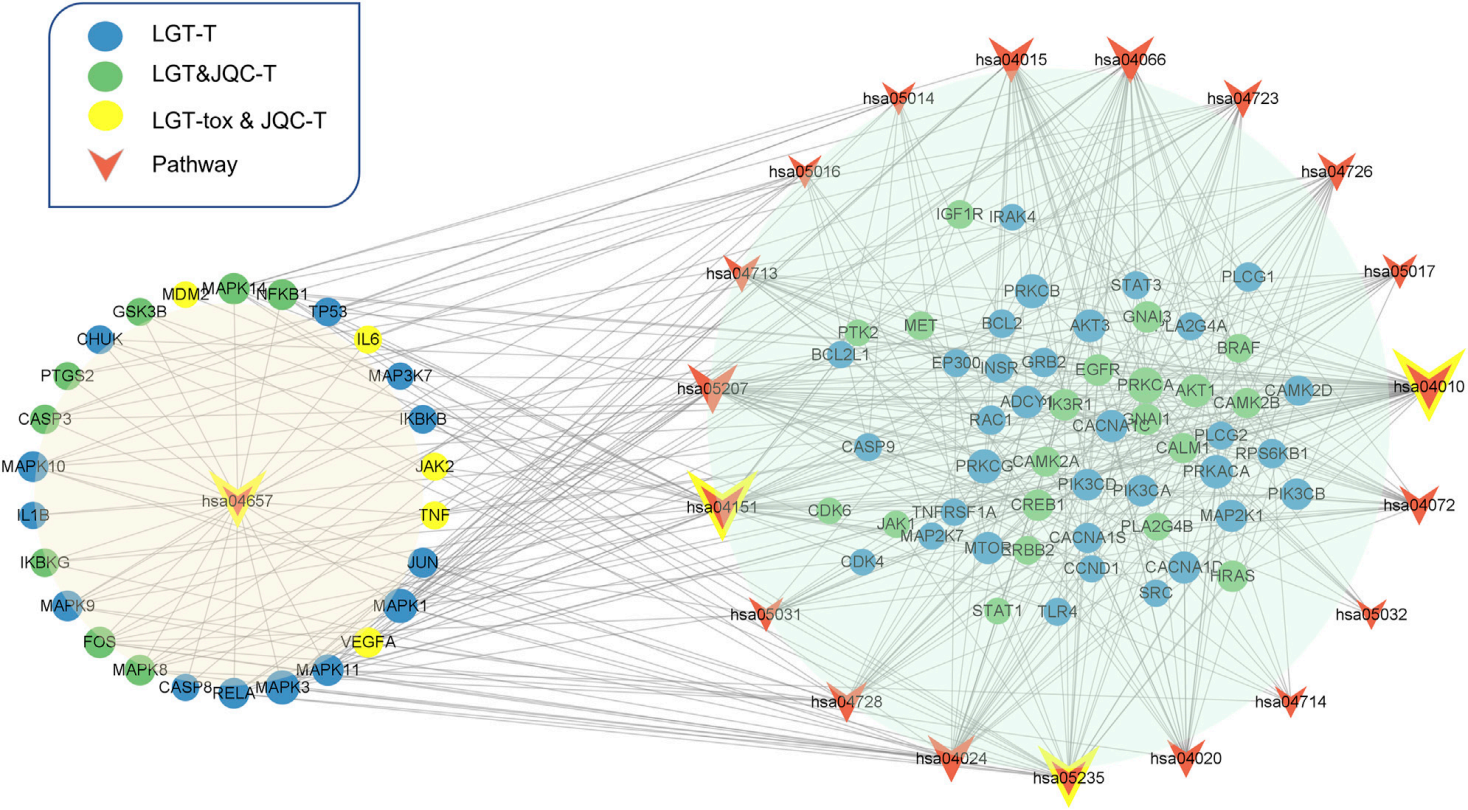
Target-pathway network of LGT-JQC. The green nodes are the common targets of LGT and JQC, and the blue represents the LGT targets, the yellow nodes are the common targets of LGT-tox and JQC, the red represents the pathways. The orange area represents the tox-related pathway, the green area represents the synergism -related pathway.

To further analyze the important pathways of the compatibility of LGT and JQC in the treatment of lung cancer, we constructed a target-pathway (T-P) network for common pathways that covered more targets, including pathways related to adverse reactions (Figure 8). The T-P network consists of 100 nodes (20 pathways and 80 targets and 437 edges). PRKCA, PRKCB, PRKCG, MAPK3, and MAPK1 had the highest degree in the T-P network. Among them, the up-regulation of PRKCA and HDGF is considered to be a negative factor for the development of lung adenocarcinoma (Jiang et al., 2019). Both PKC family and MAPK family are involved in a variety of cell signal transduction pathways and play roles in different cellular processes. They are also involved in the carcinogenesis of cancers such as lung cancer. Pathways associated with these targets are shown in addition to more prominent features. Therefore, we choose the pathway of the top four target connections in the synergistic pathway and the pathway of the highest number of target connections in toxic pathway for further study. The MAPK signaling pathway exhibits the highest number of target connections (hsa004010, degree = 44), followed PI3K-Akt pathway (hsa04020, degree = 41), Chemical carcinogenesis-receptor activation signaling pathway (hsa05207, degree = 35), PD-L1 expression and PD-1 checkpoint pathway in cancer (hsa05235, degree = 28), and toxicity target-enriched IL-17 signaling pathway (hsa05235, degree = 22), etc. MAPK pathway activity plays a key role in EGF-and IFNγ-induced PD-L1 expression in lung adenocarcinoma (Stutvoet et al., 2019). In addition, the activation of PI3K/Akt/mechanistic target of rapamycin (mTOR) pathway has become a marker of the occurrence of many cancers, and in NSCLC, the PI3K/Akt/mTOR pathway is closely related to tumorigenesis and disease progression (Tan, 2020). The activation of PD-1/PD-L1 signaling pathway is widely involved in the occurrence and development of tumors, chronic infections, and autoimmune diseases (Zhang et al., 2004). According to the literature, we can find that high-degree pathways are involved in a series of cellular physiological activities such as cell growth, development, differentiation, and apoptosis, and also have a close relationship with the occurrence of cancer. To elucidate the mechanism by which LGT works in conjunction with JQC in more detail, we conducted literature retrieval on the common pathways of high-degree.

**FIGURE 9:**
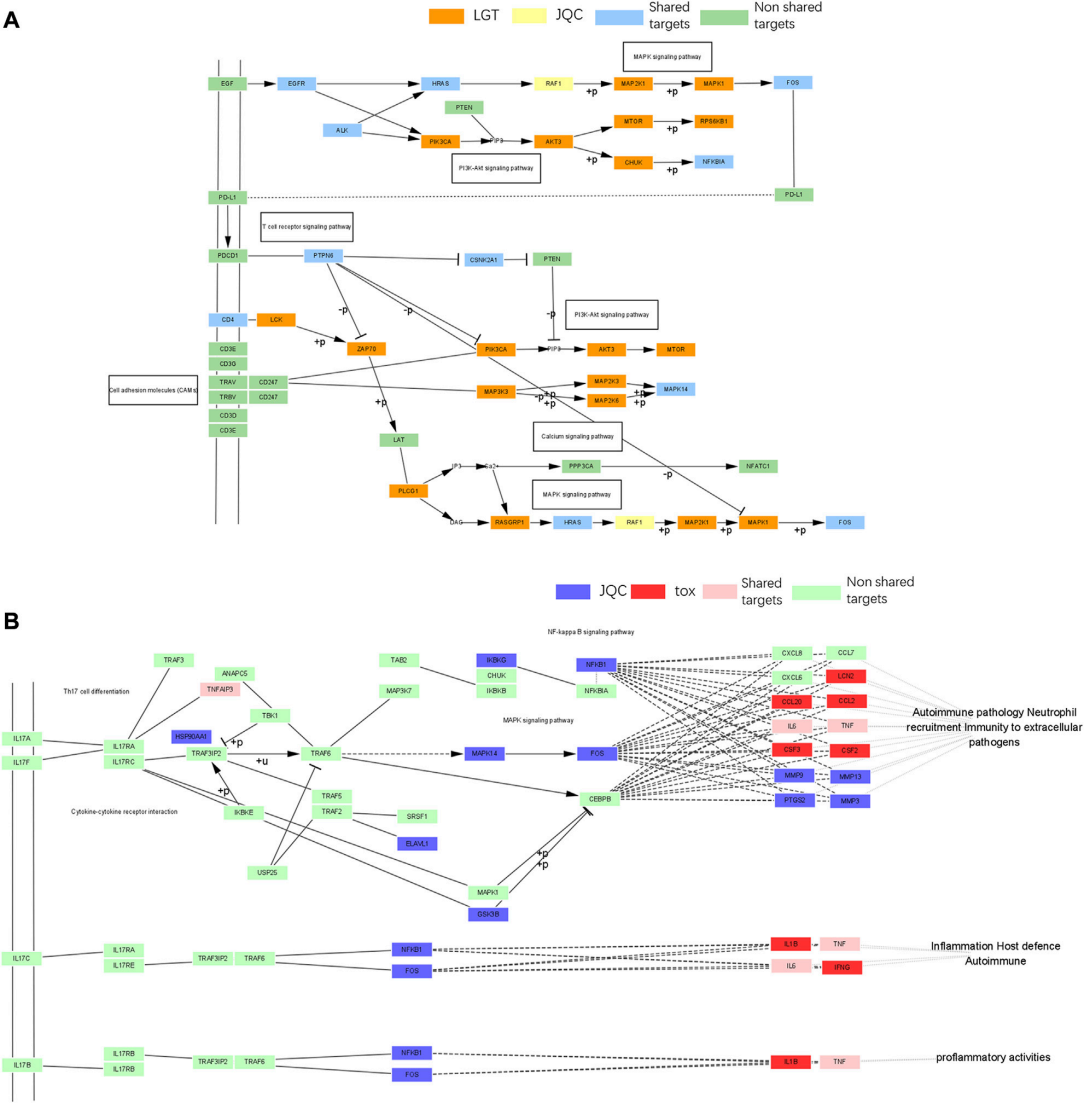
Gene enrichment pathways analysis of efficacy-toxicity network of LGT. **(A)** Gene enrichment pathways analysis of from LGT and JQC respectively; **(B)** Gene enrichment pathways analysis of JQC and LGT toxic targets.

Through published literature retrieval, we found that among the common pathways, MAPK pathway, PI3K-Akt pathway and PD-L1 expression and PD-1 checkpoint pathway in cancer had the highest correlation with lung cancer; therefore, we selected MAPK pathway, PI3K-Akt pathway, and PD-L1 expression and PD-1 checkpoint pathway in cancer to elaborate the detailed mechanism of LGT and JQC in the treatment of LC. First, we constructed an integrated signaling pathway by integrating the three pathways. The component targets of LGT and JQC were then mapped to an integrated signaling pathway (Figure 9A). The results showed that the common component targets of LGT and JQC were mainly located upstream of the comprehensive signaling pathways, such as EGFR, ALK, and CD4. The targets of JQC components were distributed downstream of the comprehensive signaling pathway, like RAF1; As for the component targets of LGT, they are present both upstream and downstream of the comprehensive signaling pathway, for example, PIK3CA, AKT3, MAP3K3, etc. This result fully demonstrated that LGT and JQC exert synergistic therapeutic effect on lung cancer by targeting different genes in the comprehensive signaling pathway as well as the common component targets.

In addition, we have found that the IL-17 pathway in the toxicity target enrichment pathway plays a key role in the pathological process of a variety of inflammatory reactions and autoimmune diseases, including the toxic and side effects of a variety of LGT such as acute renal failure and liver dysfunction. JQC covered multiple pathways related to toxicity targets including IL-17 pathway, which also indicated that JQC participated in the regulation of toxicity-related pathways such as IL-17 pathway to achieve the effect of reducing toxic and side effects on LGT. We mapped the JQC targets and the LGT toxicity targets onto the IL-17 signaling pathway (Figure 9B) to discuss the mechanisms of JQC attenuation of LGT. The results showed that the target of JQC components were mainly distributed upstream of the pathway, such as NFKB1, FOS, and MAPK14. The toxic targets of LGT were mainly distributed in the downstream of the pathways, such as LCN2, IL6, IL1B, and CSF3, and ultimately target the inflammatory host defense autoimmunity, proinflammatory activities and other inflammatory reactions and autoimmune diseases. These results suggest that JQC may attenuate the toxic and side effects of LGT by targeting upstream genes in the IL-17 pathway.

### 3.8 Experimental verification

#### 3.8.1 Experimental validation in vitro

**FIGURE 10:**
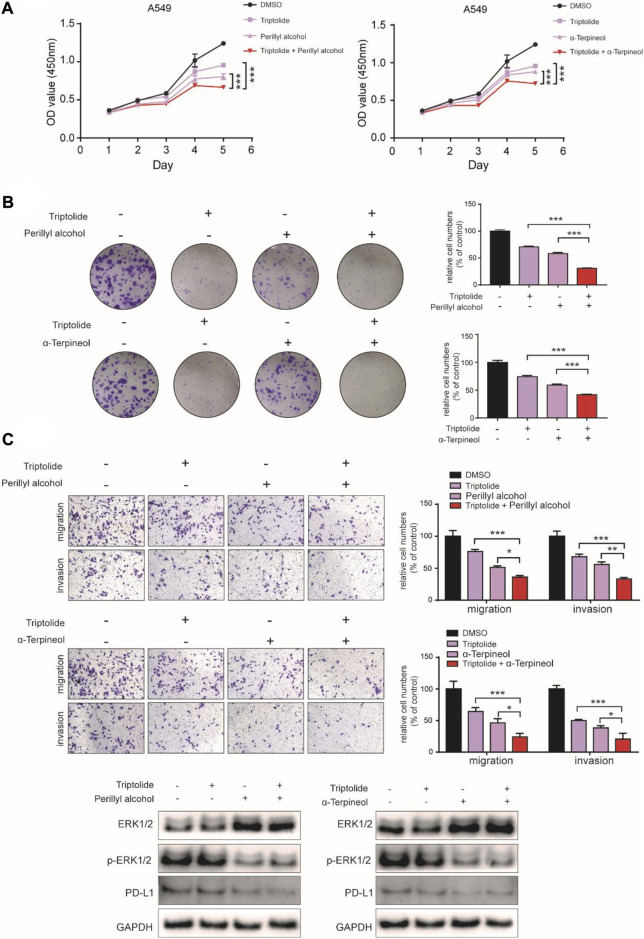
Synergistic Experimental Validation *In Vitro*. **(A–C)**: The cell proliferation, colony-formation and migration ability of the lung cancer cell A549 treated with triptolide (12.5 nM), perillyl alcohol (500 μM) and α-terpineol (500 μM) alone, or the combination of triptolide and perillyl alcohol/triptolide and α-terpineol for up to 5 days were determined by CCK-8 assay **(A)**, colony-formation assay **(B)** and transwell assay **(C)**, respectively. D Western blot assay was carried out to measure the expression of ERK1/2, p-ERK1/2 and PD-L1 in A549 cell treated with triptolide (12.5 nM), perillyl alcohol (500 μM) and α-terpineol (500 μM) alone, or the combination of triptolide and perillyl alcohol/triptolide and α-terpineol. Bars, SD; *p < 0.05, **p < 0.01, ***p < 0.001.

To further validate the reliability of the possible mechanisms of promoting efficiency of LGT and JQC in the treatment of lung cancer, the human lung cancer cell A549 was used for experiment verification *in vitro*. A549 cell was treated with triptolide (12.5 nM), perillyl alcohol (500 μM) and α-terpineol (500 μM) alone or in combination. As shown in Figures 10A,B, the combination of low-dose triptolide and perillyl alcohol or triptolide and α-terpineol were all significantly inhibited the cell viability and colony formation ability compared with inhibition by either triptolide, perillyl alcohol or α-terpineol alone. In addition, we observed the similar results from transwell assays, as the migration and invasion abilities were decreased in A549 cell exposed to the combination of triptolide and perillyl alcohol or triptolide and α-terpineol (Figure 10C). To further validate the results of the comprehensive pathway, western blot assay was employed to detect the activities of the MAPK signaling pathway and PD-L1 signaling pathway. Our data identified that the combination of triptolide and perillyl alcohol or triptolide and α-terpineol could effectively suppress MAPK signaling pathway and the expression level of PD-L1 (Figure 10D). This further verifies the accuracy of the T-P network we built. Collectively, the present results showed that although triptolide, perillyl alcohol and α-terpineol had slight antiproliferation effects individually, the combination of triptolide and perillyl alcohol or triptolide and α-terpineol could exert a synergistic effect on inhibiting cell proliferation in A549 cells.

**FIGURE 11:**
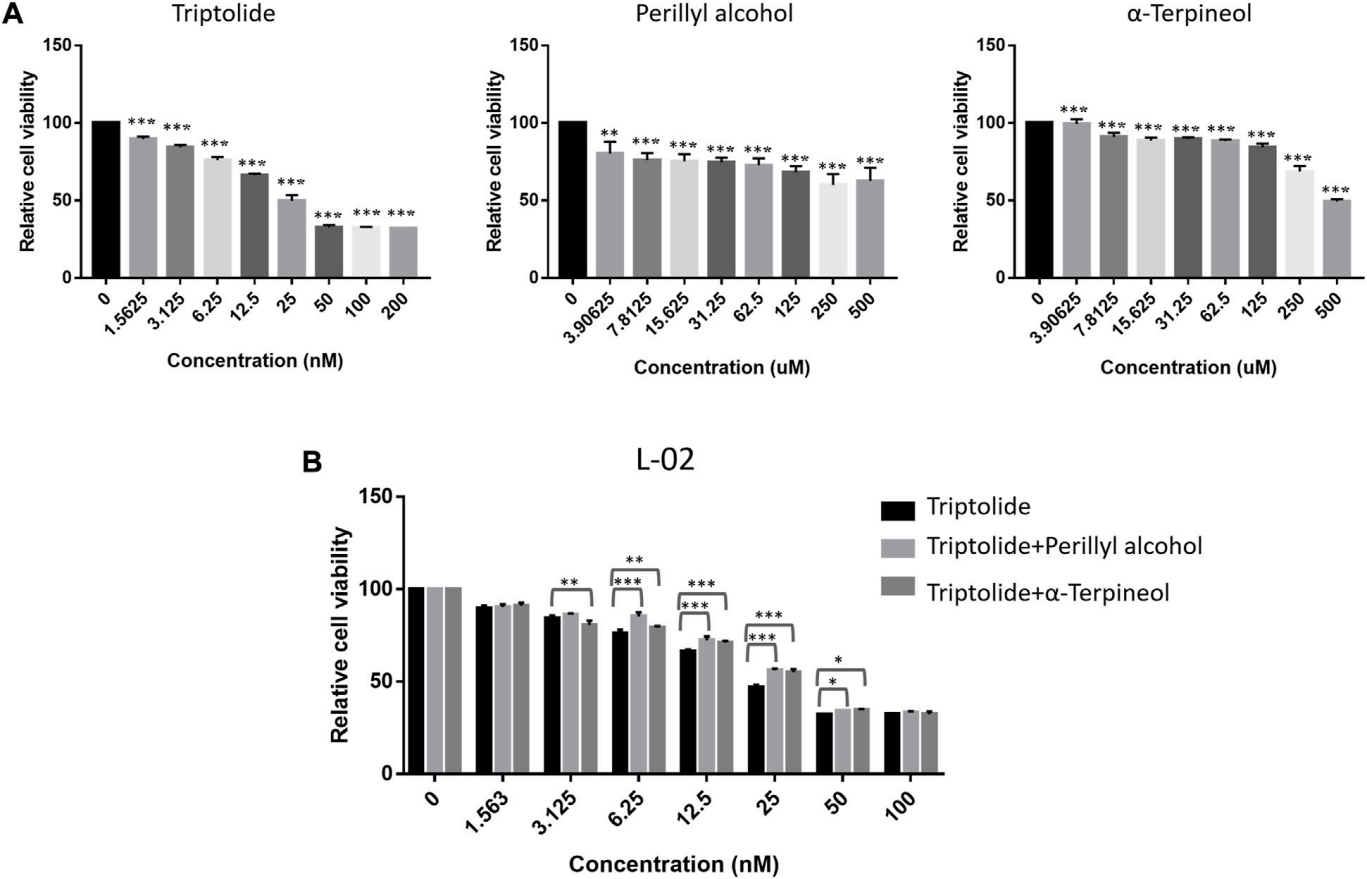
Attenuation Experimental Validation *In Vitro*. The cell proliferation ability of the human hepatocyte cell L-02 treated with triptolide, perillyl alcohol and α-terpineol alone **(A)**, or the combination of triptolide and perillyl alcohol/triptolide and α-terpineol at the ratio of 1: 500 **(B)** for 48 h were determined by CCK-8 assay. *p < 0.05, **p < 0.01, ***p < 0.001.

In addition, to validate the reducing toxicity effect of LGT and JQC in the treatment of lung cancer, the human hepatocyte cell L-02 was used for experiment verification *in vitro*. L-02 cell was treated with Triptolide, Perillyl alcohol and α-terpineol alone or in combination. As shown in Figures 11A,B, Triptolide showed obvious cytotoxicity compared with Perillyl alcohol and α-Terpineol. When the concentration of Triptolide reached 50 nM, the survival rate of L-02 cells decreased to below 30%. And then, when Triptolide is mixed with Perillyl alcohol and α-Terpineol at the ratio of 1: 500, and the drug concentration is 6.25 nM, the cell survival rate of triptolide alone is about 75%, while that of perilla alcohol combined with triptolide is about 86%, and that of α-Terpineol is about 79%. Within the concentration range of 6.25 nM–50 nM, the combination of low-dose Triptolide and Perillyl alcohol or Triptolide and α-terpineol were all significantly increased the survival rate of L-02 cells compared with the survival rate by either Triptolide, Perillyl alcohol or α-terpineol alone. Collectively, the combination of Triptolide and Perillyl alcohol or Triptolide and α-terpineol could exert a toxicity reduction effect on increasing the survival rate of L-02 cells. It indicated that the combination of LGT and JQC could exert the attenuation effect.

## 4 Discussion

In the use of TCM, the formulas play important roles in the treatment of complex diseases *via* multi-components, multi-targets and multi-pathways, and the main purpose of compatibility in formula is to promoting efficiency and reducing toxicity. As the simplest form of compatibility of TCM, herb pair is widely used in the treatment of complex diseases. Herb pair, targets and pathogenic genes form a complex intervention network. How to find the most effective intervention relationship in this intervention network and the underlying mechanisms of promoting efficiency and reducing toxicity between LGT and JQC are the keys to understand the material basis and molecular mechanism of the Chinese herb pair, and also the basis for the secondary development of TCM. At present, the research on TCM based on network pharmacology mainly focuses on decoding the potential mechanism of the efficacy of prescriptions. Through optimal model, the herbs and components with good intervention effect can be screened out, and the herbs and components with antagonistic effect and even side effect are removed, so that the herb pairs or the prescriptions is simpler and more effective (Yang et al., 2021). Compatibility rule of TCM is the foundation and basic structural unit of a prescription (SU, 2010). However, in the current stage, bioinformatics and network pharmacology models were mainly used to analyze the hidden mechanisms of prescription, while systematic biology analysis was not conducted to detection the promoting efficiency and reducing toxicity mechanisms of individual herb pairs. Here, we design network pharmacology and network toxicology methods as well as bioinformatics algorithms to screen the relationship between promoting efficiency and reducing toxicity from TCM herb pairs to explore the possible mechanism of LGT combined with JQC in the treatment of lung cancer. This integrated strategy can combine dynamic relay mode of target gene to pathogenic gene and toxicological gene. Through literature reports and experimental verification, we confirmed the accuracy of the efficacy-toxicity network. This study provided methodological reference for the secondary development of herb pairs and the development of new drugs. Our method has several advantages:

1. Network pharmacology has established a fixed analytical step in the analysis of therapeutic mechanisms. In this rule, the first step is to collect the components of the plant drugs, do ADMET screening to select the active components, then predict the target and deduce the molecular mechanism. This flowchart really solves the molecular mechanism of some formulas of TCM in treating complex diseases. Such as: Uncovering the Complexity Mechanism of Different Formulas Treatment for Rheumatoid Arthritis Based on a Novel Network Pharmacology Model (Wang et al., 2020b), Computational Network Pharmacology–Based Strategy to Capture Key Functional Components and Decode the Mechanism of Chai-Hu-Shu-Gan-San in Treating Depression (Wang et al., 2021).
2. The second step is to identify the toxicity targets from the active ingredient prediction targets, and further screen through the literature reports to retain the significant toxicity targets. However, there are also exist two problems. One is the clutter and interference of component target networks. The other is that most network pharmacology analyses ignore that the intervention of components is a cascade transmission process, specifically refers to the transmission of the intervention effect from target genes to pathogenic genes. In order to solve these two problems, we have adopted some new strategies. In the first strategy, the matrix decomposition method is used to calculate the initial influence coefficient of the component targets. Through the verification of this method, we have found that the component targets with high initial influence coefficient obtained by this method cover the majority of significant component targets of LGT, and have high coverage for the functional pathways of LGT. In the second strategy, considering that the intervention of component targets could be transmitted to the pathogenic genes through PPI network, we constructed a complex network of component-target-pathogenic genes. The network takes into account the presence of both LGT toxicity targets and JQC targets and includes the effects of the propagation. Based on this model, we constructed an efficacy-toxicity network consisting of LGT and JQC component targets, LGT toxicity targets, lung cancer pathogenic genes, and PPI network.

Through the verification of target and pathway coverage, we also found that some ineffective or weak GTCs were removed, and the significant GTCs covered most of the functional targets, further proving the effectiveness of the efficacy-toxicity network we constructed. In the efficacy-toxicity network, multiple cascade GTCs exist in the same cascade signal module. This module mainly controls the downstream genes such as CFL1, MAP2K1, ACTB, PTBP1, CSNK2A2, PPIA, KDM2A, and RAF1 to treat lung cancer through the cascade reaction of DHCR24-UBC. In this module, the first gene, DHCR24 is capable of resisting oxidative stress-induced apoptosis by scavenging hydrogen peroxide and plays a vital role in the antioxidant/anti-inflammatory cellular signaling pathway (Hou et al., 2010; Sansone et al., 2017). As an important member of the ubiquitin family, UBC has been widely reported as a target for the treatment of lung cancer. Once the regulation of ubiquitin-mediated signaling pathway is abnormal, it will lead to various clinical diseases, including tumor formation and metastasis. The transformation of protein ubiquitin in lung cancer is an important process of lung cancer development (Jin et al., 2021). In addition, in the constructed efficacy-toxicity network, we can also see the representative tumor suppressor genes such as TP53 emerging as toxicity targets, indicating that LGT has obvious toxic and side effects in the treatment of lung cancer.

Based on the propagation models of network toxicology and network pharmacology, we designed an integrated model of efficacy and toxicity which based on matrix decomposition and effect propagation, to detect the possible mechanisms of promoting efficiency and reducing toxicity of LGT and JQC in the treatment of lung cancer. Compared with other published studies, this study specifically reported the initial influence coefficients of drug component targets obtained by matrix decomposition and the construction of quantitative efficacy-toxicity network based on propagation model, which fully considers that the intervention effect of the TCM components is a cascade transmission process. Our research is a computational mining work based on pharmacological basic data, which provides a feasible scheme to reduce the verification scale for the experiment. The accurate application of this method is expected to provide a theoretical reference for the compatibility law of TCM prescriptions.

However, this study also has some limitations. First of all, we should select more targets with significant initial influence coefficients from the targets of LGT components to verify the reliability of the matrix decomposition method. In addition, the efficacy-toxicity network of component-target-pathogenic gene we constructed ignores the path loss that may occur during gene transmission. In future studies, we hope to further improve the model and try to eliminate the effect of path loss in the gene transfer process on the final result.

## Data Availability

Publicly available datasets were analyzed in this study. This data can be found here: https://portal.gdc.cancer.gov/.

## Abbreviations

LGT, *Tripterygium wilfordii* Hook. f.; JQC, *Lysimachia christinae* Hance; NSCLC, Non-small cell lung cancer; SVD, Singular value decomposition; GO, Gene Ontology; GTC, Gene Transmission Chains; LGT-ST, Significant targets for LGT; KEGG, Kyoto Encyclopedia of Genes and Genomes; PPI, Protein-protein interactions; T-P Network, Target-pathway network; C-T Network, Component-target network; TCM, Traditional Chinese medicine.
